# Altered Astrocytic Swelling in the Cortex of α-Syntrophin-Negative GFAP/EGFP Mice

**DOI:** 10.1371/journal.pone.0113444

**Published:** 2014-11-26

**Authors:** Miroslava Anderova, Jana Benesova, Michaela Mikesova, David Dzamba, Pavel Honsa, Jan Kriska, Olena Butenko, Vendula Novosadova, Lukas Valihrach, Mikael Kubista, Lesia Dmytrenko, Michal Cicanic, Lydia Vargova

**Affiliations:** 1 Department of Cellular Neurophysiology, Institute of Experimental Medicine, Academy of Sciences of the Czech Republic, Prague, Czech Republic; 2 Laboratory of Gene Expression, Institute of Biotechnology, Academy of Sciences of the Czech Republic, Prague, Czech Republic; 3 Department of Neuroscience, Charles University, 2nd Faculty of Medicine, Prague, Czech Republic; University of Modena and Reggio Emilia, Italy

## Abstract

Brain edema accompanying ischemic or traumatic brain injuries, originates from a disruption of ionic/neurotransmitter homeostasis that leads to accumulation of K^+^ and glutamate in the extracellular space. Their increased uptake, predominantly provided by astrocytes, is associated with water influx via aquaporin-4 (AQP4). As the removal of perivascular AQP4 via the deletion of α-syntrophin was shown to delay edema formation and K^+^ clearance, we aimed to elucidate the impact of α-syntrophin knockout on volume changes in individual astrocytes *in situ* evoked by pathological stimuli using three dimensional confocal morphometry and changes in the extracellular space volume fraction (α) *in situ* and *in vivo* in the mouse cortex employing the real-time iontophoretic method. RT-qPCR profiling was used to reveal possible differences in the expression of ion channels/transporters that participate in maintaining ionic/neurotransmitter homeostasis. To visualize individual astrocytes in mice lacking α-syntrophin we crossbred GFAP/EGFP mice, in which the astrocytes are labeled by the enhanced green fluorescent protein under the human glial fibrillary acidic protein promoter, with α-syntrophin knockout mice. Three-dimensional confocal morphometry revealed that α-syntrophin deletion results in significantly smaller astrocyte swelling when induced by severe hypoosmotic stress, oxygen glucose deprivation (OGD) or 50 mM K^+^. As for the mild stimuli, such as mild hypoosmotic or hyperosmotic stress or 10 mM K^+^, α-syntrophin deletion had no effect on astrocyte swelling. Similarly, evaluation of relative α changes showed a significantly smaller decrease in α-syntrophin knockout mice only during severe pathological conditions, but not during mild stimuli. In summary, the deletion of α-syntrophin markedly alters astrocyte swelling during severe hypoosmotic stress, OGD or high K^+^.

## Introduction

Cerebral edema is one of the major clinical problems accompanying pathological brain conditions such as ischemia, an abscess, trauma or the development of a tumor. Since edema formation leads to an increase in intracranial pressure, it has devastating consequences that ultimately lead to additional ischemic injury and can eventually result in brain herniation and death. Cerebral edema is caused by an excess accumulation of fluid in the brain, which is a complex process still not fully understood.

Astrocytic aquaporin-4 (AQP4), which provides a primary influx route for water during brain edema formation, is the most abundant water channel in the CNS located mostly in the endfeet of perivascular astrocytes and adjacent to blood vessels, thus acting at the CNS-blood interface. In addition, in the glia limitans and subependymal astrocytes and ependymal cells AQPs play a role in water transport at CNS–cerebrospinal fluid (CSF) interface [1]–[3]. Such pattern of AQP4 expression suggests that they enable water flow into and out of the CNS. Experiments with AQP4-null mice have shown that AQP4 is a key element in the formation of brain edema [4] because its knockout markedly reduces water permeability in astrocytes isolated from AQP4-null mice [5]. The site-specific anchoring of AQP4 is dependent on the α-syntrophin protein, which is a member of the dystrophin complex, and its deletion leads to the disruption of the polarized subcellular expression of AQP4 in the perivascular membranes [6], which makes these mice a valuable tool for studying cerebral edema.

There are several studies showing that in α-syntrophin knockout mice, AQP4 is not present in the perivascular and subpial endfeet of astrocytes, which was proven by electron microscopy as well as immunohistochemical labeling [1], [7], [8]. According to these studies, AQP4 expression is not altered in other parts of the astrocytic membranes or in endothelial cells in these mice. Moreover, Western blots comparing the cerebellum and neocortex of wild-type and α-syntrophin knockout mice showed that the deletion of α-syntrophin does not cause any changes in the tissue content of AQP4, indicating that the AQP4 channels are only redistributed within the astrocytic membranes upon the deletion of α-syntrophin [1], [8]. These findings are supported by immunogold analysis, which shows increased AQP4 labeling in non-endfeet membranes of astrocytes in α-syntrophin knockout mice when compared to wild type mice [6]. The weakly rectifying K^+^ channel Kir4.1 was shown to be associated with the dystrophin complex via α-syntrophin, which suggests that Kir4.1 and AQP4 may be co-associated via their respective interaction with α-syntrophin [9]. Studies with α-syntrophin knockout mice have revealed a slight decrease in astrocytic perivascular Kir4.1; nevertheless, the reduction of Kir4.1 was much lower when compared to AQP4 and was not consistent among experimental animals [10].

The influence of α-syntrophin on edema formation is not so straightforward. On the one hand, the deletion of α-syntrophin, accompanied by the downregulation of AQP4 in the perivascular membranes of astrocytes, delays/diminishes the development of brain edema in a model of acute hyponatremia as well as after water intoxication, middle cerebral artery occlusion, terminal ischemia/anoxia and hypoosmotic stress [6], [11], [12]. On the other hand, in models simulating vasogenic brain edema, such as intraparenchymal fluid infusion, focal cortical freeze injury and tumor cell implantation [2], the deletion of AQP4 led to augmented intracranial pressure and brain water content due to the impaired clearance of intraparenchymal fluid. Alpha-syntrophin also plays an important role in astrocytic K^+^ spatial buffering as its deletion prolongs K^+^ clearance when compared with wild type mice [10], [12]. The reduced capacity for K^+^ clearance along with a decrease in the size of the extracellular space due to edema formation further results in increased vulnerability to epileptic seizures [10], [13], [14].

The observed changes of edema formation in α-syntrophin knockout mice mentioned above were studied utilizing diffusion-weighted magnetic resonance imaging, the real-time iontophoretic method or by quantifying infarct volume [12]; however, all of these approaches enable the study of cerebral edema formation solely on the tissue level. The aim of the present work was to extend these findings by elucidating the effect of α-syntrophin deletion in swelling of individual astrocytes under various pathological conditions, and by clarifying the contribution of astrocytic soma and processes to total astrocytic volume changes. For this purpose we crossbred α-syntrophin knockout mice with GFAP/EGFP mice in order to visualize single astrocytes, which were monitored for volume changes under different conditions utilizing 3D confocal morphometry [15]. To further identify the pathways by which astrocytes in α-syntrophin knockout mice compensate/regulate their swelling, we employed the reverse transcription quantitative PCR (RT-qPCR) technique to detect differences in gene expression of the main astrocytic channels and transporters.

## Materials and Methods

### Transgenic mice

For quantifying astrocyte volume changes, we used transgenic GFAP/EGFP mice in which the enhanced green fluorescent protein (EGFP) is expressed under the control of the promoter for human glial fibrillary acidic protein (GFAP) [16]. Fluorescently labeled astrocytes enable direct measurement of single astrocyte volume changes in acute brain slices using 3D-confocal morphometry as described previously [15], [17]. To elucidate the impact of α-syntrophin knockout on astrocytic swelling, we generated double transgenic mice (GFAP/EGFP/α-Syn^−/−^ mice) by crossbreeding GFAP/EGFP mice (FVB/N) and mice with a genetic deletion of α-syntrophin (C57BL/6) [18]. All procedures involving the use of laboratory animals were performed in accordance with the European Communities Council Directive 24 November 1986 (86/609/EEC) and animal care guidelines approved by the Institute of Experimental Medicine ASCR Animal Care Committee.

### Identification of double transgenic GFAP/EGFP/α-Syn^−/−^ mice

The α-syntrophin deletion as well as its impact on the AQP4 expression pattern in double transgenic mice was confirmed using PCR, western blotting and immunohistochemistry.

To validate the α-syntrophin deletion in crossbred GFAP/EGFP/α-Syn^−/−^ mice, PCR analysis of genomic DNA isolated from the tail was employed, and the following primers were used for α-syntrophin: forward 5′-GGTGGCGACTGGCTCTGCTG-3′, reverse 5′-AGCGCTTCCTGGCAGCTGTGG-3′; for neomycin (Neo): forward 5′-CAAATTAAGGGCCAGCTCATTCCTCC-3′, reverse 5′-ACAGGAGCCCAGTCTTCAATCCAGG-3′. DNA extraction from tail biopsies and PCR amplification were performed using a REDExtract-N-Amp Tissue PCR Kit (Sigma-Aldrich, St. Louis, MO, USA). PCR products were separated on a 1.5% agarose gel and visualized with SYBR Safe DNA Gel Stain (Molecular Probes, Invitrogen, Carlsbad, CA, USA). Besides lacking the gene for α-syntrophin, DNA samples isolated from α-Syn^−/−^ and GFAP/EGFP/α-Syn^−/−^ expressed the gene for neomycin resistance (*Neo*), which was inserted into the gene construct as a positive selective marker during homologous recombination.

The absence of α-syntrophin protein was verified using western blot analysis. Cortical tissue samples were isolated from GFAP/EGFP mice, α-Syn^−/−^ mice or GFAP/EGFP/α-Syn^−/−^ mice and homogenized using an ultrasound homogenizator in Tris buffer (pH 6.8) containing 10% glycerol and 1% sodium dodecyl sulphate (SDS). Total protein content in the homogenates was determined by the Micro BCA protein assay kit (Thermo Fisher Scientific, Rockford, IL, USA). Tissue homogenates were heated at 100°C for 5 min with 0.5% dithiothreitol. Equal amounts of proteins were loaded and separated on a 10% SDS-polyacrylamide gel and subsequently electro-transferred to a nitrocellulose membrane with a TE 70XP Semi-Dry Transfer unit (Hoefer, Holliston, MA, USA). Membranes were blocked with 5% non-fat dry milk in PBS-Tween buffer (0.05% Tween) for 1 hour at room temperature. Incubation with the primary rat anti-Syntrophin alpha 1 antibody (AB11187, Abcam, Cambridge, UK), diluted in PBS containing 1% non-fat dry milk, 0.05% Tween and 0.1% NaN_3_, was performed at 4°C overnight, followed by a 2 hour incubation with the secondary anti-rabbit IgG antibody conjugated with peroxidase (Sigma-Aldrich, St. Louis, MO, USA) at RT. SuperSignal West Pico Chemiluminiscent Substrate (Thermo Fisher Scientific) was used to visualize the separated proteins.

The impact of α-syntrophin deletion on AQP4 localization in the cortex of GFAP/EGFP/α-Syn^−/−^ mice was compared with original knockouts (α-Syn^−/−^ mice) and GFAP/EGFP mice using immunohistochemistry. The animals were deeply anesthetized with pentobarbital (100 mg/kg, i.p.) and transcardially perfused with 20 ml of saline with heparin (2500 IU/100 ml; Zentiva, Prague, Czech Republic) followed by 20 ml of 4% paraformaldehyde solution. Brains were dissected out, post-fixed in 4% paraformaldehyde for 3 hours and placed stepwise in solutions with gradually increasing sucrose concentrations (10%, 20%, 30%) for cryoprotection. Coronal slices (30 µm thick) were prepared using a microtome (HM 400, Thermo Fisher Scientific, Waldorf, Germany). The slices were incubated in blocking solution - 5% ChemiBLOCKER (Chemicon, Temecula, CA, USA) and 0.5% Triton X-100 in PBS - for 1 hour. The blocking solution was also used as a diluent for the antibodies. The slices were incubated overnight at 4°C with the primary rat anti-AQP4 antibody (AB3594, Chemicon, Temecula, CA, USA), followed by a 2-hour incubation with goat anti-rabbit IgG secondary antibody conjugated with Alexa-Fluor 594 (Molecular Probes, Invitrogen, Carlsbad, CA, USA) at room temperature. A Zeiss 510DUO LSM spectral confocal microscope equipped with Ar/HeNe lasers was used for immunohistochemical analysis. All chemicals were purchased from Sigma-Aldrich (St. Louis, MO, USA) unless otherwise stated.

### Solutions

The slices were kept in artificial cerebrospinal fluid (aCSF), which contained (in mM): NaCl 122.0, KCl 3.0, CaCl_2_ 1.5, MgCl_2_ 1.3, Na_2_HPO_4_ 1.25, NaHCO_3_ 28.0, D-glucose 10.0. The pH was adjusted to 7.4 by gassing the aCSF with 95% O_2_ and 5% CO_2_. Osmolality was confirmed to be 300±5 mOsm/kg with a vapour pressure osmometer (Vapro 5520, Wescor Inc., Logan, USA). A solution simulating the extracellular conditions during ischemia, hypoxia and hypoglycemia (oxygen-glucose deprivation, OGD) was prepared by gassing the aCSF with 5% O_2_, 5% CO_2_ and 90% N_2_, without adding D-glucose as described previously [19]. Its osmolality was confirmed to be 300±5 mOsm/kg. Hyperosmotic aCSF was prepared by the addition of sucrose to normal aCSF at a final concentration of 44 mM (aCSF_hyper+sucrose_) or by the addition of mannitol at a final concentration of 50 mM (aCSF_hyper+mannitol_). The osmolality of both hyperosmotic solutions was confirmed to be 350±5 mOsm/kg. Hypoosmotic solutions (aCSF_H-50_, aCSF_H-100_) were prepared by reducing the NaCl concentration to 98.0 mM or to 58 mM, and their osmolarities were 250 and 205 mOsm/kg, respectively. Solutions modeling hyperkalemia (aCSF_K+10_, aCSF_K+50_) were prepared by the addition of 10 mM or 50 mM KCl together with a concurrent reduction of NaCl concentration. Their osmolality was confirmed to be 300±5 mOsm/kg.

### Preparation of tissue slices

Astrocyte volume changes *in situ* were studied in the cortex of 1-month-old transgenic mice; diffusion parameter measurements were performed *in situ* and *in vivo* in the cortex of 3–4-month-old transgenic mice. For *in situ* recordings, animals (both males and females) were anesthetized with isoflurane and decapitated. The brains were quickly dissected out and placed into cold (4°C) aCSF. Transversal slices (400 µm) were prepared using a HM 650 V microtome with a vibrating blade (MICROM Int. GmbH, Waldorf, Germany). Slices were kept in aCSF at room temperature (23–25°C).

For *in vivo* experiments, 3-month-old mice were anesthetized with isoflurane (1.5% in a gas mixture of 35% O_2_/65% N_2_O) administered by a facemask, and their heads were fixed in a stereotaxic holder. Body temperature was maintained at 37°C by a heating pad. The somatosensory cortex was partially exposed by a burr hole 1.5 mm caudal from bregma and 1.5 mm lateral from the midline, and the dura was carefully removed. The exposed brain tissue was bathed in warm (37°C) aCSF. An experimental model of terminal ischemia/anoxia *in vivo* was evoked by cardiac arrest induced by the intraperitoneal administration of 0.5 ml of saturated MgCl_2_ (4.7M) [20]. Electrocardiographic recording was used to monitor heart arrest, which occured 60–90 s after i.p. injection.

### Measurement of extracellular K^+^ concentration

The extracellular potassium concentration ([K^+^]_o_) in the mouse cortex *in vivo* during terminal ischemia/anoxia was measured by double-barreled K^+^-sensitive microelectrodes, as described in detail previously [21]. The tip of the K^+^-selective barrel of the microelectrode was filled with the liquid ion-exchanger Corning 477317 (currently available as IE 190 from World Precision Instruments, Sarasota, Florida, USA) and back-filled with 0.5 mM KCl; the reference barrel contained 150 mM NaCl. Electrodes were calibrated in a sequence of solutions containing 0.5, 1, 2, 3, 4, 6, 8, 12, 16, 24, 32, 48, 64, 96 and 128 mM KCl, with a background of NaCl with decreasing concentration to keep the ionic strength of the solution constant (150 mM). The data were fitted to the Nikolsky equation to determine the electrode slope and interference. Based on these electrode characteristics, the measured voltage was converted to extracellular concentrations.

### Measurements of the extracellular space diffusion parameters

To determine the extracellular space (ECS) diffusion parameters in the motor and somatosensory cortices (layers II–IV) – ECS volume fraction α (α = ECS/total tissue volume), tortuosity λ (λ^2^ = *D/ADC*, where *D* is the free diffusion coefficient and *ADC* is the apparent diffusion coefficient in the brain tissue), and non-specific concentration-dependent or independent uptake *k′* – the real-time iontophoretic (RTI) method, described in details previously [22], was used. Briefly, the principle of the method is the administration of an extracellular marker such as the tetramethylammonium ion (TMA^+^, Mw = 74.1 Da) into the extracellular space by iontophoresis and the measurement of its local concentration with a TMA^+^-selective microelectrode (TMA^+^-ISM). Double-barreled TMA^+^-ISMs were prepared similarly as K^+^-ISMs; the procedure has been described previously [21]. The tip of the ion-sensitive barrel was filled with a liquid ion exchanger for K^+^ (Corning 477317 or IE 190 from World Precision Instruments, Sarasota, Florida, USA) that is also highly sensitive to TMA^+^ ions; to ensure that the ion-selective microelectrode (ISM) was primarily sensitive to TMA^+^, the rest of the barrel was backfilled with 150 mM TMA^+^ chloride. The reference barrel contained 150 mM NaCl. The electrodes were calibrated using the fixed-interference method before each experiment in a series of solutions of 150 mM NaCl+3 mM KCl (or 100 mM NaCl+53 mM KCl for measurements during ischemia) with the addition of the following concentrations of TMA chloride: 0.25, 0.5, 1, 2, 4, 8 and 16 mM. The slope and interference values obtained by calibration and estimated by the VOLTORO program [22] were used by the same program to calculate the real concentration of TMA^+^ during the experiment. An electrode array was made by gluing a TMA^+^-ISM to an iontophoretic micropipette with a tip separation of 100–150 µm. Typical iontophoresis parameters were 20 nA bias current (continuously applied to maintain a constant transport number) and a +180 nA current step with a 24 sec duration to generate the diffusion curve. TMA^+^ diffusion curves were first recorded in 0.3% agar (Sigma-Aldrich, Steinheim, Germany) dissolved in a solution of 150 mM NaCl, 3 mM KCl and 1 mM TMACl, in which by definition α = 1, λ = 1 and *k′* = 0 (free-diffusion values). The diffusion curves obtained in agar were analyzed to yield the electrode transport number (*n*) and the free TMA^+^ diffusion coefficient (*D*) by curve fitting according to a modified diffusion equation using the VOLTORO program. Diffusion curves were then generated in the somatosensory cortex at a depth of 450–500 µm or in the middle of the cortical slices (at a depth of 200 µm) at regular intervals of 5 min. Knowing *n* and *D*, the values of α, λ and *k′* can be obtained from the diffusion curves.

### Three-dimensional confocal morphometry

For cell volume quantification, we used 3D-confocal morphometry, which was developed in our institute [17], [23]–[25]. Briefly, the brain slices of GFAP/EGFP or GFAP/EGFP/α-Syn^−/−^ mice were placed in a perfusion chamber and perfused either with aCSF or modified aCSF solutions to evoke astrocyte volume changes. All experiments were performed at room temperature (23–25°C). EGFP was excited with an Ar laser set at 488 nm, and the emitted signal was recorded over the range of 510–552 nm using a TD488/543/633 filter. To minimize photo-bleaching, the laser intensity was always held at 25%. The signal was recorded using a Leica TCS SP system confocal microscope (Leica, Germany) with a water immersion 40× (0.8) HCX APO Leica objective (Leica, Germany). The microscope was equipped with a perfusion chamber in which a perfusion rate of ∼5 ml/min was maintained by a PCD 31.2 pump (Kouřil, Czech Republic). All data were saved with Leica Confocal Software (Leica, Germany).

Protoplasmic astrocytes from the vicinity of blood vessels were selected in the motor and somatosensory cortices (layers I–IV) based on their bright fluorescence and numerous bushy processes [16], [26]. As the intensity of EGFP expression, which is driven by GFAP promotor, varies in astrocytes, the size of the selected astrocytes varied as well.

Astrocyte volume changes were determined from 3D images of individual astrocytes. The astrocytes were recorded as a set of confocal images with an image size of 1024×1024 pixels. Every 3D image of the cell was sectioned into 70–80 consecutive 2D images with a uniform spacing of 1 µm. The scanning time for 70–80 2D images was ∼150 s. The cell surface was found in each image using an edge-detecting algorithm, and the area of the image surrounded by the edge was calculated for each layer. The values of cell volume for individual cells were obtained by integrating the values of the edge length and area from all images in a set. Image processing and morphometric measurements were performed using the program CellAnalyst developed in the Department of Cellular Neurophysiology, Institute of Experimental Medicine, Prague, Czech Republic. Our previous studies demonstrated that during repetitive exposure of EGFP-labeled astrocytes to an excitatory laser beam their total cell volume decreases in a linear fashion (Fig. S1A, B). We showed that the decreases in the morphometric data are linear and do not depend on the time interval between individual scans [17], [24]. Therefore, a fluorescent signal decrease in the obtained morphometric values was first compensated for by using a time-independent algorithm based on data from three control measurements prior to the application of a pathological stimulus (Fig. S1C). The average run down of fluorescence between two consecutive scans was 4.3±0.5% (n = 47 cells).

### Statistics

Astrocyte volume changes are presented as the mean ± S.E.M. Statistical significance was evaluated by a two-tailed unpaired Student's *t*-test. Differences between the groups were considered statistically significant when the p value was equal to- or lower than 0.05.

### Quantitative RT-PCR

RT-qPCR was used to quantify the gene expression levels of astrocytic membrane channels and transporters in EGFP-labeled astrocytes isolated from three GFAP/EGFP/α-Syn^−/−^ and three GFAP/EGFP mice.

#### Preparation of cortical astrocytes

30–40-day-old mice were used in the experiment. Cortex collection, the preparation of the cell suspensions, as well as the collection of EGFP^+^ cells were performed as previously described [27]. The maximal number of cells (typically 40 000 cells) was collected into a 1.5-ml Eppendorf tube containing 500 µl RLT buffer with β-mercaptoethanol (Qiagen, Germany). Tubes with collected cells were immediately frozen at −80 C.

#### RNA extraction and cDNA synthesis

An RNeasy Micro Kit (Qiagen, Germany) was used for RNA extraction according to the manufacturer's recommendation. SuperScript III RT (Life Technologies, Czech Republic) was employed for cDNA synthesis. Extracted RNA containing 0.5 mM dNTP (Promega, Germany), 1 mM oligo(dT15) (Invitrogen) and 1 mM random hexamers (Invitrogen) was incubated at 70°C for 5 min. The mix was cooled down on ice, and 50 mM Tris–HCl, 75 mM KCl, 3 mM MgCl_2_, 5 mM dithiothreitol, 40 U RNaseOut and 200 U SuperScript III (all Invitrogen; final concentrations) were added to a final volume of 20 µl. Reverse transcription (RT-PCR) was performed at 25°C for 5 min, 50°C for 60 min, 55°C for 10 min and terminated by heating to 75°C for 15 min. The RT-PCR step was carried out in duplicate. Five µl of cDNA was used for preamplification.

#### Preamplification

Each reaction contained 25 µl of iQ Supermix (BioRad, Czech Republic), 5 µl of a mix of all primers (Table 1; final concentration 25 nM each), 5 µl of non-diluted cDNA, and water added to a final volume of 50 µl. The temperature profile was 95°C for 3 min followed by 18 cycles of amplification (95°C for 20 s, 57°C for 4 min and 72°C for 20 s on a Biorad CFX96). The protocol was validated on samples from two animals. In addition, technical replicates of preamplification were performed for each sample. The expression of all genes was measured in preamplified and non-preamplified samples. Low standard deviations (≤0.5) of the differences between preamplified and non-preamplified samples confirmed the protocol's reproducibility.

**Table 1:** Sequences of primers used for PCR.

Gene	PubMed ID	Forward and reverse primer (5′>3′)	Length (bp)	Exon-exon spanning	Efficiency	Protein
***Clcn1***	NM_013491.2	F:GCAGAGCGAAGGTTGAAGG R:TCTACGAAGGCAAAGGACTCAG	128	no	0.90	ClC1
***Clcn2***	NM_009900.2	F:TGCCAATGTCTTCCTTACTCTG R:ATTCGGTAGGTGCTGCTATC	198	yes	0.96	ClC2
***Glul***	NM_008131.3	F:CGCAAAGACCCCAACAAG R:ATTCCTGCTCCATTCCAAAC	135	yes	0.96	Glutamine synth.
***Trpv4***	NM_022017.3	F:AGAGACAAGTGGCGTAAGTT R:GCTGATAGTAGGCGGTGAG	100	no	0.94	TRPV4
***Kcnk1***	NM_008430.2	F:GGGAAATTGGAATTGGGACTTCA R:TGCCGATGACAGAGTAGATGAT	134	yes	0.97	TWIK-1
***Kcnk2***	NM_010607.2	F:ATTCGTATCATCTCCACCATCATC R:CACACCACAGGCTTGTAGAA	209	yes	0.84	TREK-1
***Kcnk9***	NM_001033876.1	F:TGACTACTATAGGGTTCGGC R:GAATCGCAGGACCACAAGAT	149	no	0.94	TASK-3
***Kcnk10***	NM_029911.4	F:CACTGTGGCTATCTCCTTAACC R:GGCTGAGGCGGTGTAATC	111	no	0.95	TREK-2
***Kcnj3***	NM_004981.1	F:ATGGACTAGATGACATTAGCACAA R:AGGAACTGAACTTATTCGTTGGA	164	no	0.97	Kir3.1
***Kcnj10***	NM_001039484.1	F:AACTTGGGAGATTGAGATATGATATA R:AAGTCTGAATACTTCCTTCTGTAC	129	no	0.94	Kir4.1
***Kcnj16***	NM_010604.3	F:CCTGTGTCTCCTCTTGAAGG R:TGTGCTTAGGTGATACAATACGG	158	no	0.93	Kir5.1
***Aqp1***	NM_007472.2	F:CTGGCTGCGGTATCAACC R:GGATGAAGTCATAGATGAGCACTG	132	yes	0.96	AQP1
***Aqp4***	NM_009700.2	F:CGGCATCCTCTACCTGGTCACA R:GCCAGCGGTGAGGTTTCCAT	82	yes	0.96	AQP4
***Aqp9***	NM_022026.2	F:GAAGGATGGAGTGGTTCAAGTTC R:TGGCACGGATACAAATGGTTT	137	yes	1.00	AQP9
***Gja1***	NM_010288.3	F:TCTCACCTATGTCTCCTCCT R:GCTGGCTTGCTTGTTGTA	86	no	0.99	Cx43
***Gjb6***	NM_001010937.1	F:TAAGAATAAGCCTGCACGATGGA R:CCACTAGGATCATGACTCGGA	130	no	0.93	Cx30
***Slc1a3***	NM_148938.3	F:ATCGTCCTGCCTCTCCTCTAC R:GTCCACACCATTGTTCTCTTCCA	159	yes	0.98	GLAST
***Slc1a2***	NM_011393.2	F:GAGAGCAGGCACACTTACA R:GTAGAAGAATCTGGATACCGAAGG	159	no	0.92	GLT-1
***Gfap***	NM_010277.3	F:ACAGACTTTCTCCAACCTCCA R:CAGGGCTCCATTTTCAATC	159	yes	1.00	GFAP
***Gfap-delta***	NM_010277.3	F:ATGTGTCTCAGTTGTGAAGGTCTA R:TGGAAGGATGGTTGTGGATTCT	108	no	0.96	GFAP (splice δ)
***Atp1a1***	NM_144900.1	F:CCTCTGCTTCGTGGGTCTTATC R:TCGCTGTGATTGGATGGTCTC	128	yes	0.97	NaKATPase alfa1
***Atp1a2***	NM_178405.3	F:GAAGGACTTGGCTTGCTAA R:GCTACGAGGACGAGGATA	107	no	0.95	NaKATPase alfa2
***Nkcc1***	NM_009194.3	F:ATTACGGTGGCTAACACTGG R:GCATCTCTAAGAACTAATGGTTGA	98	no	0.94	NKCC1
***Kcc1***	NM_009195.2	F:TACAAGTACATCGAGTACCAAGG R:GTCTAACTTAAGCAGCACCAGGAG	159	yes	0.95	KCC1
***Kcc3***	NM_133648.2	F:CATTCCAGGGTTGGCTAGTG R:TGTGACCGAGGGAAAGAAGA	190	yes	0.92	KCC3

*Clcn1* and *Clcn2* code voltage-gated chloride channels ClC1 and ClC2; *Glul* encodes glutamine synthetase (GS); *Trpv4* encodes transient receptor potential vanilloid 4 channels TRPV4; *Kcnk1*, *Kcnk2*, *Kcnk9* and *Kcnk10* code two pore domain K^+^ channels TWIK-1, TREK-1, TASK-3 and TREK2; *Kcnj3*, *Kcnj10* and *Kcnj16* code inwardly rectifying K^+^ channels Kir3.1, Kir4.1 and Kir5.1; *Aqp1*, *Aqp4* and *Aqp9* code water channels AQP1, AQP4 and AQP9; *Gja1* and *Gjb6* code connexin43 and connexion 30; *Slc1a3* and *Slc1a2* code glutamate transporters GLAST and GLT-1, *Gfap* and *Gfap-delta* code glial fibrillary acidic protein and its δ splice variant. *Atp1a1* and *Atp1a2* code NaK-ATPases, *Nkcc1*, *Kcc1* and *Kcc3* code Na-dependent and independent K^+^/Cl^−^ co-transporters.

#### Quantitative real-time PCR

A Biorad CFX384 (Biorad, Czech Republic) was used for all qPCR measurements. To each reaction (10 µl) containing 5 µl iQ SYBR Green Supermix (BioRad) and 300 nM of each primer (EastPort, Czech Republic) was added 2 µl of 20-fold diluted preamplified cDNA. The temperature profile was 95°C for 3 min followed by 40 cycles of amplification (95°C for 15 s, 60°C for 20 s and 72°C for 20 s). All samples were analyzed by melting curve analysis. Primers were designed using BeaconDesigner (version 7.91, Premier Biosoft International) as described previously [15]. The primer sequences are shown in Table 1.

#### Data processing and analysis

qPCR data were pre-processed and analysed in GenEx software (MultiD, version 5.3). Briefly, Cq values were corrected to different efficiencies (Table 1), normalized with qPCR repeats, normalized with different numbers of cells and normalized with the geometric average of the two most stable reference genes - *B2m* and *Ywhaz*. These reference genes were chosen from a panel of 12 reference genes - Reference Gene Panel for Mouse (Tataa, Sweden). The algorithms GeNorm and NormFinder were used for the evaluation [28], [29]. Normalized values were further transformed into relative quantities, and a log2 scale was applied. Pre-processed data were analysed with basic statistics (average expression, standard deviation etc.). Student's t-test was used to compare the expression of individual genes between three GFAP/EGFP/α-Syn^−/−^ and three GFAP/EGFP mice.

## Results

### Generation of double transgenic GFAP/EGFP/α-Syn^−/−^ mice

**Figure 1:**
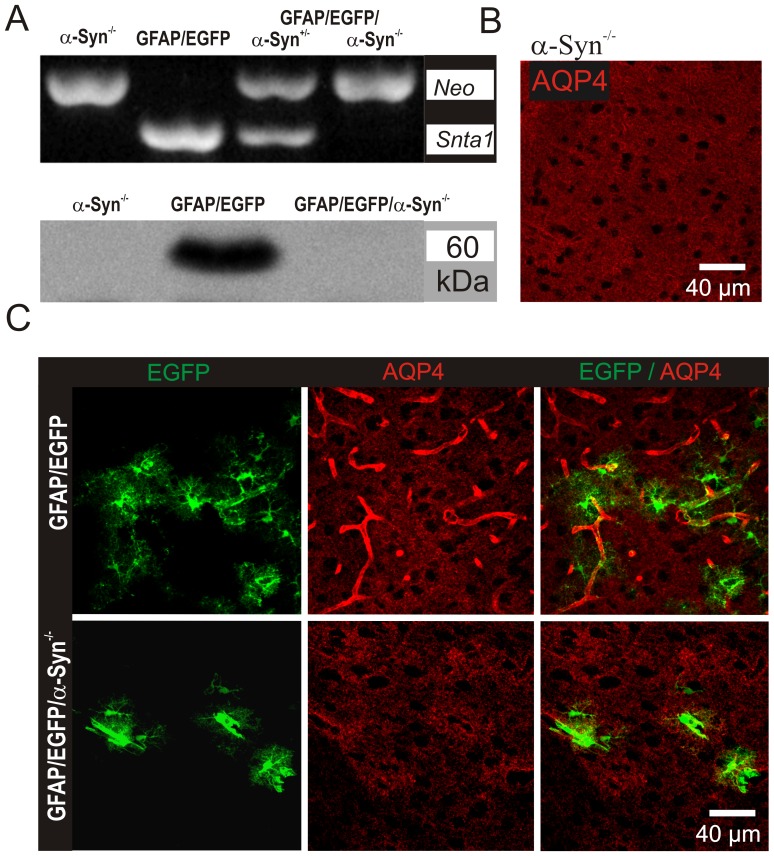
Characterization of double transgenic GFAP/EGFP/α-Syn^−/−^ mice. (**A**) PCR of tail genomic DNA isolated from α-Syn^−/−^ mice, GFAP/EGFP mice, or double transgenic GFAP/EGFP/α-Syn^−/−^ mice. Mice lacking the *Snta1* gene coding α-syntrophin also express *Neo*, the gene of neomycin resistance, inserted in the gene construct as a positive selectable marker during homologous recombination (top). Western blot analysis detecting α-syntrophin in cortical tissue isolated from α-Syn^−/−^ mice, GFAP/EGFP mice, or GFAP/EGFP/α-Syn^−/−^ mice (bottom). (**B**) Immunohistochemical staining for aquaporin 4 (AQP4) in cortical slices isolated from α-Syn^−/−^ mice, (**C**) GFAP/EGFP mice or GFAP/EGFP/α-Syn^−/−^ mice. Note that α-syntrophin deletion is accompanied by the loss of AQP4 expression on the astrocytic membranes contacting blood vessels.

To elucidate the impact of α-syntrophin knockout on astrocyte volume changes evoked by pathophysiological stimuli, we generated double transgenic mice (GFAP/EGFP/α-Syn^−/−^ mice) by crossbreeding GFAP/EGFP mice with mice having genetically deleted α-syntrophin (α-Syn^−/−^). The α-syntrophin deletion as well as its impact on the AQP4 expression pattern in the double transgenic mice was confirmed using PCR, Western blotting and immunohistochemistry (Fig. 1). Analyses of DNA samples isolated from α-Syn^−/−^ or GFAP/EGFP/α-Syn^−/−^ animals confirmed that besides the lack of the gene for α-syntrophin, they also contained a sequence for neomycin resistance (*Neo*), which was inserted into the gene construct as a positive selective marker during homologous recombination (Fig. 1A top). Furthermore, western blot analyses confirmed the absence of α-syntrophin in both α-Syn^−/−^ as well as GFAP/EGFP/α-Syn^−/−^ mice (Fig. 1A bottom). The impact of α-syntrophin deletion on AQP4 localization in the cortex of GFAP/EGFP/α-Syn^−/−^ mice was assessed by immunohistochemistry, by comparing cortical slices of newly generated double transgenic mice with α-Syn^−/−^ mice and GFAP/EGFP mice (Fig. 1B, C, Fig. S2A). We found that the pattern of AQP4 staining in double transgenic GFAP/EGFP/α-Syn^−/−^ mice is similar to that described in α-Syn^−/−^ mice [11]. GFAP/EGFP mice were used as controls for all experiments. Since we subsequently used cortical slices for evaluating changes in the diffusion parameters and astrocyte volume, we also tested the possibility of AQP4 redistribution in the astrocyte membrane of EGFP/GFAP mice in response to slice preparation or the long-lasting incubation of slices in aCSF [30]. Nevertheless, we found no differences in AQP4 immunohistochemistry between slices that were prepared from brains following the trans-cardial perfusion of the animals with paraformaldehyde (Fig. 1) and those acutely prepared for diffusion parameter measurements or 3D confocal morphometry. In addition, 90 minute-incubation of slices in aCSF had no effect on AQP4 distribution as well (Fig. S2).

### Changes in diffusion parameters in double transgenic mice in various experimental models of cell swelling

We employed the RTI method with TMA^+^ to demonstrate that the cortical slices of our newly generated transgenic mice display similar characteristics with respect to changes in the diffusion parameters observed in cortical slices of α-Syn^−/−^ mice during their exposure to pathological stimuli. In agreement with our previous study [12], which compared α-Syn^+/+^ and α-Syn^−/−^ mice, diffusion measurements in coronal slices *in situ* as well as in the somatosensory cortex *in vivo* revealed significantly lower basal values of ECS volume fraction α in GFAP/EGFP mice in comparison with GFAP/EGFP/α-Syn^−/−^ animals, with no significant difference between these animal groups in the basal values of tortuosity λ or non-specific uptake *k′* (Table S1, S2).

### Effects of hypotonic stress and elevated K^+^ on ECS diffusion parameters *in situ*

**Figure 2:**
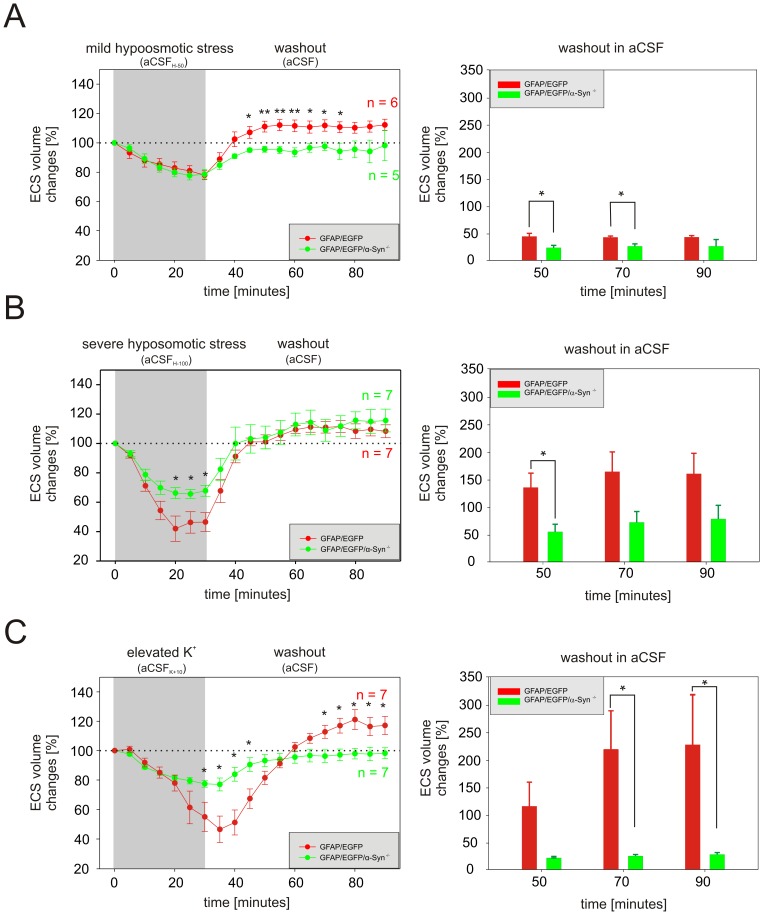
The effect of hypotonic stress or elevated K^+^ on the ECS volume *in situ*. The left side: The control values of all experiments were set to 100%, and the relative changes of the values of the extracellular volume fraction α were calculated at 5 min intervals during a 30 min application and a subsequent 60 min washout of mild (**A**) or severe (**B**) hypotonic stress or 10 mM K^+^ (**C**). Each data point represents mean ± S.E.M. The right side: volume regulation during washout at 20 min intervals is expressed as changes in the values reached in the 30^th^ minute of application, set as 0%. Asterisks indicate significant (*, p<0.05) and very significant (**, p<0.01) differences between GFAP/EGFP and GFAP/EGFP/α-Syn^−/−^ mice.

A 30 min application of solutions simulating mild (aCSF_H-50_) or severe (aCSF_H-100_) hypotonic stress resulted in a significant decrease in ECS volume fraction α due to cell swelling in both GFAP/EGFP and GFAP/EGFP/α-Syn^−/−^ mice (Table S1). Since the basal values of α in GFAP/EGFP and GFAP/EGFP/α-Syn^−/−^ mice differ, we evaluated both the absolute as well as the relative changes of the ECS volume fraction. To estimate the relative change, the control values of all experiments were set to 100%, and the relative changes in α during perfusion were calculated. During superfussion with aCSF_H-50_, we did not find any significant difference between GFAP/EGFP/α-Syn^−/−^ and GFAP/EGFP mice in either the absolute values of α or in its relative changes (Table S1, Fig. 2A). However, exposure to more severe hypotonic stress revealed that the deletion of α-syntrophin results in a smaller decrease in α in both absolute as well as relative values (Table S1, Fig. 2B). During washout following aCSF_H-50_ perfusion, the absolute and relative values of α overshot the control values (100%) in the cortex of GFAP/EGFP mice. The observed overshoot in the ECS volume in the cortex of GFAP/EGFP mice is a physiological response, which is related to the prolonged effect of the regulatory volume decrease (RVD) mechanisms, evoked by cell swelling [31]–[33]. Since no overshoot was observed in the cortex of GFAP/EGFP/α-Syn^−/−^ mice (Table S1, Fig. 2A, B), we may conclude that the changes in α during washout are smaller in the cortex of GFAP/EGFP/α-Syn^−/−^ mice. Interestingly, during washout following aCSF_H-100_ application the values of α obtained in the cortex of GFAP/EGFP/α-Syn^−/−^ mice were not significantly different from those in GFAP/EGFP mice. To further analyse ECS volume changes during the washout period, they were expressed as a percent of volume increase/decrease in relation to the value of α reached in the 30^th^ minute of application, which was set as 0%. At the beginning of washout after either mild or severe hypotonic stress, smaller α changes were observed in the cortex of GFAP/EGFP/α-Syn^−/−^ mice than in GFAP/EGFP mice (Fig. 2A, B, right) however, the magnitude of α changes after 90 minutes of washout was comparable in both types of mice. Changes in λ or *k′* evoked by aCSF_H-50_ or aCSF_H-100_ were not significantly different between the animal groups (Table S1).

A decrease in α observed during a 30 min superfusion with aCSF_K+10_ was significantly smaller in GFAP/EGFP/α-Syn^−/−^ mice than in GFAP/EGFP animals in both absolute as well as relative values (Table S1, Fig. 2C). During washout, the values of α overshot the control values in GFAP/EGFP mice, but not in GFAP/EGFP/α-Syn^−/−^ animals (Fig. 2C). Relative ECS volume changes during washout, related to the value of α reached in the 30^th^ minute of application, showed a smaller α changes in GFAP/EGFP/α-Syn^−/−^ mice when compared to GFAP/EGFP mice (Fig. 2C). A small increase in λ evoked by 10 mM K^+^ did not differ between the two animal groups (Table S1).

### Effect of ischemia/anoxia *in vivo*

**Figure 3:**
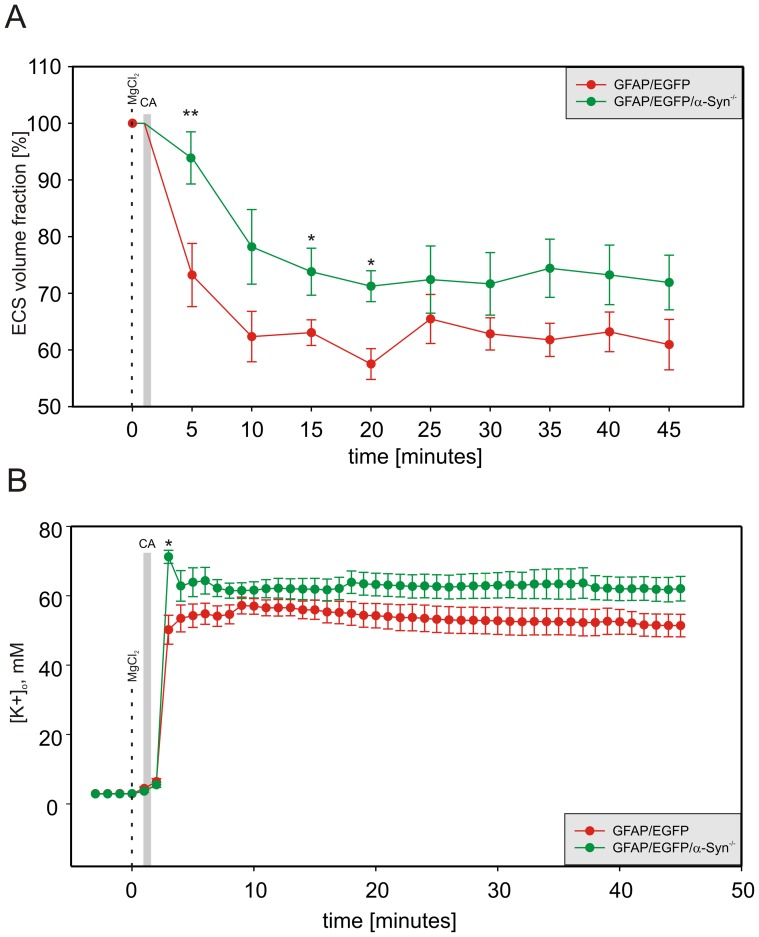
Relative changes in the ECS volume fraction and [K^+^]_o_ evoked by terminal ischemia/anoxia *in vivo*. Each data point represents mean ± S.E.M, calculated at 5 min intervals for ECS volume changes and each minute for [K^+^]_o_ changes. The relative changes in the values of the ECS volume fraction α (**A**) after the onset of terminal ischemia/anoxia were significantly smaller and slower in GFAP/EGFP/α-Syn^−/−^ mice than in GFAP/EGFP controls, but the final values did not differ. In contrast, the steep rise in [K^+^]_o_ levels (**B**) was higher and faster in GFAP/EGFP/α-Syn^−/−^ mice compared to GFAP/EGFP animals, with no difference in the final values. The gray bar indicates cardiac arrest (CA) and the dashed line indicates MgCl_2_ injection. Asterisks indicate significant (*, p<0.05) and very significant (**, p<0.01) differences between GFAP/EGFP and GFAP/EGFP/α-Syn^−/−^ mice.

Terminal ischemia/anoxia due to cardiac arrest evoked by the administration of MgCl_2_ resulted in dramatic changes in both ECS volume fraction α and tortuosity λ. The minimal absolute values of α reached after cardiac arrest were larger in GFAP/EGFP/α-Syn^−/−^ mice than in GFAP/EGFP animals, and the time course of relative changes of α indicated smaller changes in GFAP/EGFP/α-Syn^−/−^ animals compared to GFAP/EGFP animals (Table S2, Fig. 3). Surprisingly, λ in GFAP/EGFP/α-Syn^−/−^ mice reached a higher maximum than in GFAP/EGFP animals (Table S2), suggesting the larger formation of additional diffusion barriers. A steep rise of [K^+^]_o_ immediately after the onset of terminal ischemia/anoxia was faster and reached its maximum in GFAP/EGFP/α-Syn^−/−^ mice in about 3 min after cardiac arrest. In GFAP/EGFP mice, the [K^+^]_o_ increase was gradual, reaching the highest point at the 9^th^ minute, with the maximum value significantly lower than in GFAP/EGFP/α-Syn^−/−^ animals (Table S2, Fig. 3).

All these results indicate that the responses of GFAP/EGFP/α-Syn^−/−^ and GFAP/EGFP animals to selected experimental models of cell swelling are similar to those observed in α-Syn^−/−^ and α-Syn^+/+^ mice, respectively [12]. We can therefore assume that GFAP/EGFP/α-Syn^−/−^ mice display similar characteristics as α-Syn^−/−^ animals and that the results obtained by the following analyses of astrocytic volume changes are valid for both GFAP/EGFP/α-Syn^−/−^ and α-Syn^−/−^ mice.

### The effect of α-syntrophin deletion on astrocyte volume changes evoked by osmotic stress

AQP4s, which are the main water channels in astrocytes, participate significantly in regulatory volume processes. The deletion of α-syntrophin, which results in the redistribution of AQP4 molecules in astrocytic membranes [6], may thus lead to the impairment of such regulatory processes. To test this hypothesis, we employed two models– hypoosmotic and hyperosmotic stress.

**Figure 4:**
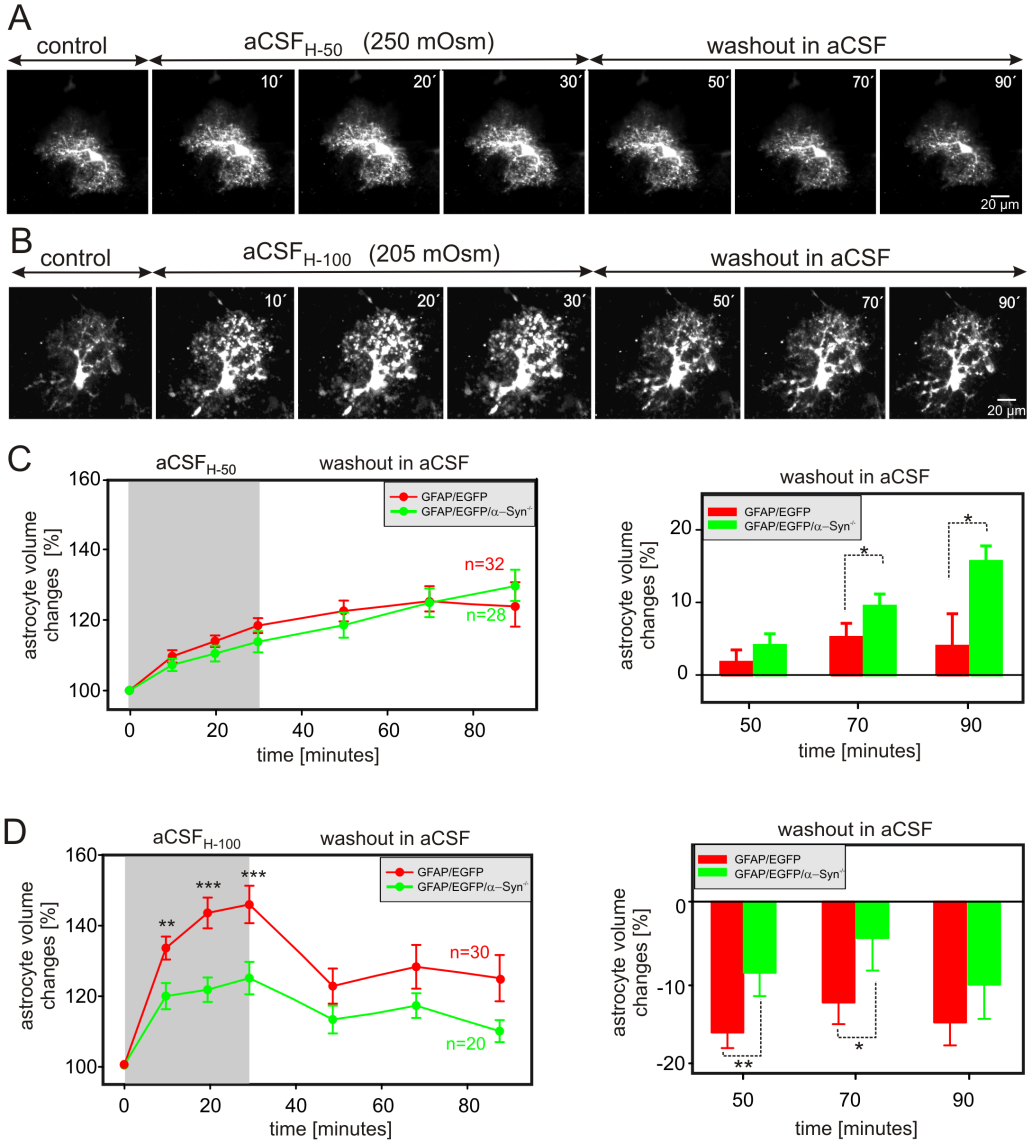
Astrocytic volume changes in the cortex of GFAP/EGFP and GFAP/EGFP/α-Syn^−/−^ mice during hypoosmotic stress. (**A, B**) Superimposed confocal images of EGFP-labeled cortical astrocytes from GFAP/EGFP/α-Syn^−/−^ mice obtained in aCSF (control), during a 30-minute exposure to hypoosmotic stress of 250 mOsm/kg (aCSF_H-50_; **A**) or 205 mOsm/kg (aCSF_H-100_; **B**) and during a 60-minute washout. The astrocytic volume was quantified every 10 minutes during exposure to hypoosmotic stress and every 20 minutes during washout. (**C, D**) Time-dependent changes in the total astrocytic volume in GFAP/EGFP (red) and GFAP/EGFP/α-Syn^−/−^ mice (green) during a 30-minute exposure to aCSF_H-50_ (**C left**) or to aCSF_H-100_ (**D left**) and during a 60-minute washout. The changes of astrocytic volume during washout were evaluated in each individual cell and expressed as an average percent of cell volume increase/decrease related to the maximal volume after 30 minutes of aCSF_H-50_ (**C right**) or aCSF_H-100_ (**D right**) exposure; these values were set as 0%. Note that α-syntrophin deletion results in smaller astrocyte swelling only during aCSF_H-100_ application and in smaller volume changes following mild hypoosmotic stress. Asterisks indicate significant (*, p<0.05), very significant (**, p<0.01) and extremely significant (***, p<0.001) differences between GFAP/EGFP and GFAP/EGFP/α-Syn^−/−^ mice.

The impact of hypoosmotic stress on astrocyte volume in GFAP/EGFP/α-Syn^−/−^ mice was tested using two types of hypotonic aCSF solutions – aCSF_H-50_ (250±5 mOsm/kg) and aCSF_H-100_ (205±5 mOsm/kg). The astrocyte volume changes were quantified during a 30-minute exposure to aCSF_H-50_ or aCSF_H-100_ and following a 60-minute washout in isosmotic aCSF (300±5 mOsm/kg). The cell volume was determined by 3D-confocal morphometry every 10 minutes during the exposure to hypoosmotic solutions and every 20 minutes during washout (Fig. 4A, B). Surprisingly, the exposure of cortical slices to aCSF_H-50_ evoked comparable astrocytic swelling in both GFAP/EGFP and GFAP/EGFP/α-Syn^−/−^ mice, while no differences were obvious during the washout period (Fig. 4C). After 30 minutes of aCSF_H-50_ application, astrocytic volume increased to 118.4±2.6% (n = 32 cells) in GFAP/EGFP and to 113.8±2.9% (n = 28 cells) in GFAP/EGFP/α-Syn^−/−^ mice. During washout, astrocyte volume in both mouse strains further increased reaching 127.7±6.9 and 132.0±4.7% of the original volume, respectively. Since the changes in cell volume of individual astrocytes in either GFAP/EGFP or GFAP/EGFP/α-Syn^−/−^ mice are not uniform and since the degree of astrocyte swelling or shrinkage in response to a pathological stimulus might play a role in astrocyte volume changes the during washout period, the magnitude of the changes in astrocytic volume following aCSF_H-50_ application was evaluated in each individual cell and expressed as a percent of cell volume increase/decrease related to the maximal volume reached after 30 minutes of hypoosmotic stress, which was set as 0%. Values above 0 indicate an additional increase in astrocyte volume (astrocyte swelling), while those below 0 represent a decrease in astrocyte volume (astrocyte shrinkage). Such analysis revealed that during washout following mild hypoosmotic stress the volume of astrocytes lacking α-syntrophin further increased when compared to astrocytes from GFAP/EGFP mice.

On the other hand, marked dissimilarities between GFAP/EGFP mice and those lacking α-syntrophin were found in astrocytic swelling in response to aCSF_H-100_. The exposure of slices to aCSF_H-100_ disclosed that α-syntrophin deletion results in a smaller swelling of astrocytes. A 30-minute application of aCSF_H-100_ increased astrocytic volume to 145.6±5.3% (n = 30 cells) in GFAP/EGFP mice and only to 124.7±4.6% (n = 20 cells) in GFAP/EGFP/α-Syn^−/−^ mice (Fig. 4D left). In addition, the changes in astrocytic volume following aCSF_H-100_ application were evaluated in each individual cell and expressed as a percent of cell volume increase/decrease related to the maximal volume reached after 30 minutes of hypoosmotic stress, which was set as 0% (Fig. 4D right). Astrocytes in GFAP/EGFP/α-Syn^−/−^ as well as GFAP/EGFP mice decreased their volume during washout and the magnitude of such a volume decrease (shrinkage) was smaller in astrocytes from GFAP/EGFP/α-Syn^−/−^ mice during the first 40 minutes of washout.

**Figure 5:**
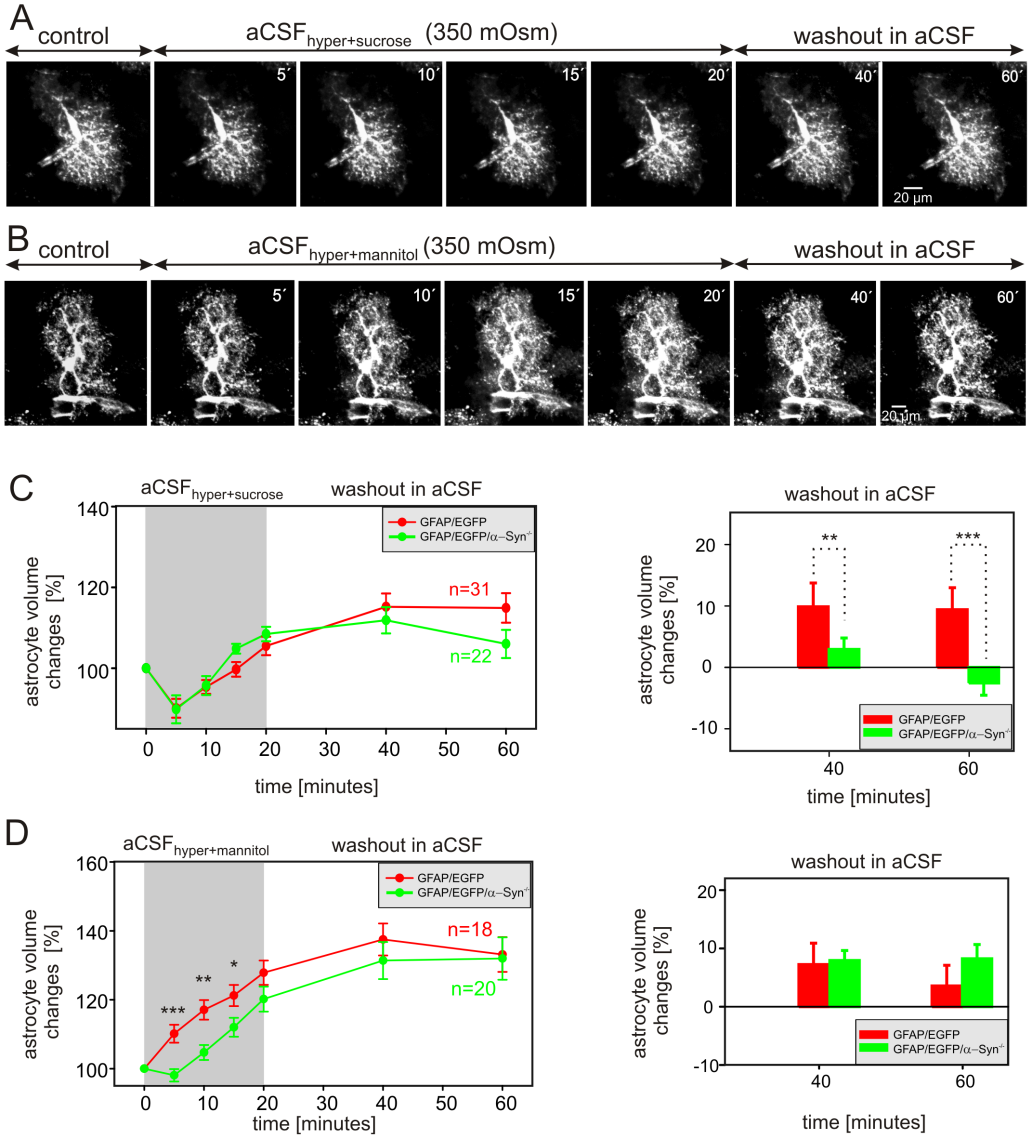
Astrocytic volume changes in the cortex of GFAP/EGFP and GFAP/EGFP/α-Syn^−/−^ mice during hyperosmotic stress. (**A, B**) Superimposed confocal images of EGFP-labeled astrocytes in the cortex of GFAP/EGFP/α-Syn^−/−^ mice recorded in aCSF (control), during a 20-minute exposure to hyperosmotic stress of 350 mOsm/kg using sucrose-containing aCSF (aCSF_hyper-sucrose_, **A**) or mannitol-containing aCSF (aCSF_hyper-mannitol_, **B**), and during a 40-minute washout. The astrocytic volume was quantified after every 5 minutes during exposure to hyperosmotic stress and every 20 minutes during washout. Time-dependent changes in the total astrocytic volume in GFAP/EGFP (red) and GFAP/EGFP/α-Syn^−/−^ mice (green) during a 30-minute exposure to aCSF_hyper-sucrose_ (**C left**) or during a 30-minute application of aCSF_hyper-mannitol_ (**D left**) and during a 60-minute washout. The changes of astrocytic volume during washout were evaluated in each individual cell and were expressed as an average percent of cell volume increase/decrease related to the maximal volume after 20 minutes of aCSF_hyper-sucrose_ (**C right**) or aCSF_hyper-mannitol_ application (**D right**); these values were set as 0%. Asterisks indicate significant (*, p<0.05), very significant (**, p<0.01) and extremely significant (***, p<0.001) differences between GFAP/EGFP and GFAP/EGFP/α-Syn^−/−^ mice.

The effect of hyperosmotic stress was examined using modified aCSF solutions containing either 44 mM sucrose (aCSF_hyper+sucrose_) or 50 mM mannitol (aCSF_hyper+mannitol_) in order to obtain an osmolality of 350 mOsm/kg. Astrocyte volume changes were quantified during a 20-minute application of hyperosmotic solution and during 40-minute washout. The cell volume was determined every 5 minutes during hyperosmotic stress and every 20 minutes during washout (Fig. 5A, B). The exposure of astrocytes to aCSF_hyper+sucrose_ did not reveal any differences between GFAP/EGFP and GFAP/EGFP/α-Syn^−/−^ mice. In both experimental groups a 5-minute exposure to aCSF_hyper+sucrose_ led to an astrocytic volume decrease of ∼10%, while further exposure to hyperosmotic stress resulted in a volume increase to 105.5±2.3% (n = 31) and 108.5±1.8% (n = 22), respectively (Fig. 5C left). After switching aCSF_hyper+sucrose_ to normal aCSF, an additional increase in astrocytic volume was observed in GFAP/EGFP mice, while the cell volume of astrocytes of GFAP/EGFP/α-Syn^−/−^ mice did not change during washout. In addition, the changes in astrocytic volume following aCSF_hyper+sucrose_ application were evaluated in each individual cell and expressed as a percent cell volume increase/decrease related to the maximal volume after 20 minutes of hyperosmotic stress (set as 0%, Fig. 5C right). Astrocytes in the cortex of GFAP/EGFP mice further increased their volume during the washout period; such a volume increase is related to the prolonged effect of regulatory volume increase (RVI) mechanisms [34]–[36], which results in additional astrocytic swelling even during washout. Of note, astrocytes from GFAP/EGFP/α-Syn^−/−^ mice did not show such a volume increase (swelling) during washout. An interesting finding was obtained during hyperosmotic stress induced by the application of aCSF_hyper+mannitol_ (Fig. 5D left). Instead of the expected cell shrinkage, astrocytes from GFAP/EGFP mice started to swell immediately after the onset of hyperosmotic stress, and after 15 minutes the average astrocyte volume was 121.2±3.1% (n = 18), while the swelling of astrocytes from GFAP/EGFP/α-Syn^−/−^ mice was smaller (112.0±2.7%, n = 20). During washout the astrocytes from both types of transgenic mice further increased their volume, and these changes were comparable (Fig. 5D right).

### Volume changes in α-syntrophin-deficient astrocytes evoked by increased extracellular K^+^ concentration

Since α-syntrophin deletion also impairs efficient K^+^ uptake from the extracellular space [10], we determined the impact of increased extracellular K^+^ concentrations on astrocytic volume changes in GFAP/EGFP/α-Syn^−/−^ mice.

**Figure 6:**
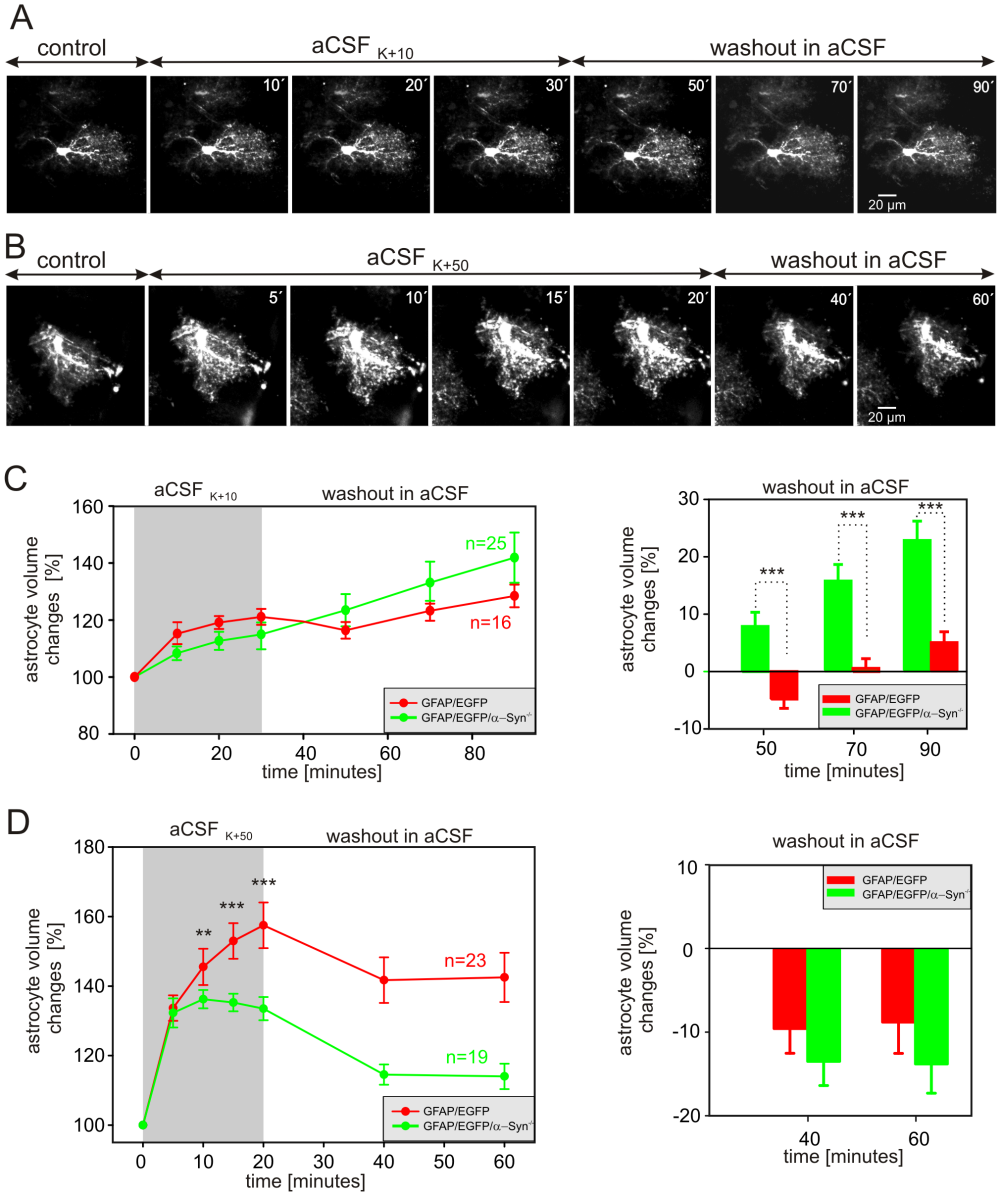
Astrocytic volume changes evoked by increased extracellular K^+^ concentration in the cortex of GFAP/EGFP and GFAP/EGFP/α-Syn^−/−^ mice. (**A, B**) Superimposed confocal images of EGFP-labeled astrocytes in the cortex of GFAP/EGFP/α-Syn^−/−^ mice recorded in aCSF (control), during exposure to aCSF containing 10 mM K^+^ (aCSF_K+10_, **A**) or 50 mM K^+^ (aCSF_K+50_, **B**), and during washout. The astrocytic volume was quantified every 10 minutes during exposure to hyperkalemia and every 20 minutes during washout. Time-dependent changes in the total astrocytic volume in GFAP/EGFP (red) and GFAP/EGFP/α-Syn^−/−^ mice (green) during a 30-minute exposure to aCSF_K+10_ (**C left**) or a 20-minute exposure to aCSF_K+50_ (**D left**) and during washout. The changes of astrocytic volume during washout were evaluated in each individual cell and were expressed as an average percent of cell volume increase/decrease related to the maximal volume after 30 minutes of aCSF_K+10_ (**C right**) or after 20 minutes of aCSF_K+50_ application (**D right**); these values were set as 0%. Asterisks indicate very significant (**, p<0.01) and extremely significant (***, p<0.001) differences between GFAP/EGFP and GFAP/EGFP/α-Syn^−/−^ mice.

Initially, we studied the effect of 10 mM [K^+^]_o_, which is a typical [K^+^]_o_ measured in the extracellular space during epileptiform activity [37]. The astrocyte volume changes were quantified during a 30-minute exposure to aCSF_K+10_ and following a 60-minute washout. The cell volume was determined every 10 minutes during exposure to aCSF_K+10_ and every 20 minutes during washout (Fig. 6A). Thirty-minute exposure to aCSF_K+10_ did not reveal any differences in astrocytic volume changes between GFAP/EGFP and GFAP/EGFP/α-Syn^−/−^ mice (Fig. 6C left). The astrocyte volume increased gradually in both GFAP/EGFP and GFAP/EGFP/α-Syn^−/−^ mice, reaching 120.6±2.8% (n = 16) and 114.9±5.2% (n = 25), respectively. During washout, the volume of astrocytes from GFAP/EGFP mice initially decreased by 7% during the first 20 minutes of washout, then started to increase again (to 128.5% in the 90^th^ minute), while the volume of GFAP/EGFP/α-Syn^−/−^ astrocytes further increased, up to 141.9%. Furthermore, the astrocytic volume changes following aCSF_K+10_ application were expressed as a percent of cell volume increase/decrease related to the maximal volume after 30 minutes of exposure to 10 mM K^+^ (set as 0%, Fig. 6C right). Such analysis revealed that astrocytes in the cortex of GFAP/EGFP mice show only a slight volume increase after a 60 minute-washout, while astrocytes from GFAP/EGFP/α-Syn^−/−^ mice further increase their volume and the magnitude of their volume changes is greater than that observed in astrocytes of GFAP/EGFP mice.

To simulate extracellular K^+^ concentrations reached in the ECS during brain ischemia [38], we used aCSF containing 50 mM K^+^. Here, the astrocyte volume changes were quantified every 5 minutes during a 20-minute exposure to aCSF_K+50_ and every 20 minutes during a 40-minute washout (Fig. 6B). We found that α-syntrophin deletion markedly affected the astrocyte swelling induced by exposure to aCSF_K+50_ (Fig. 6D left), while it did not influence astrocyte volume changes during washout (Fig. 6D right). In GFAP/EGFP/α-Syn^−/−^ mice, the astrocyte volume increased within the first ten minutes of exposure to aCSF_K+50_, and after reaching its maximum (136.3±2.6%), it started to decline to 133.3±2.5% (n = 19). On the other hand, in GFAP/EGFP mice the astrocyte volume was increasing continuously during the entire exposure to aCSF_K+50_, eventually reaching 157.5±6.5% (n = 23) of the initial volume. During washout the decrease in astrocytic volume was comparable in both groups of animals (Fig. 6D right).

### The influence of α-syntrophin deletion on astrocytic swelling following oxygen-glucose deprivation

**Figure 7:**
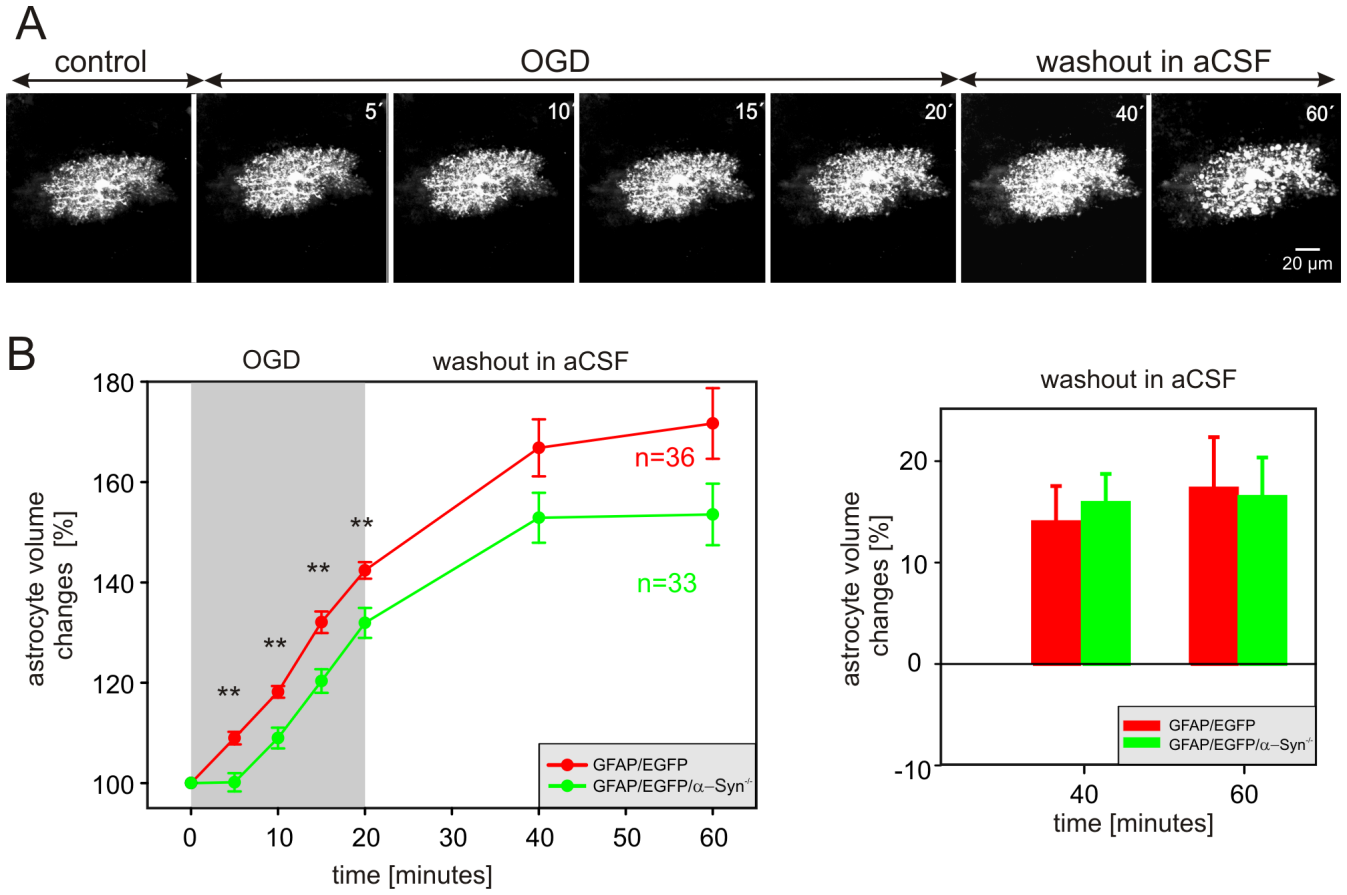
Astrocytic volume changes evoked by oxygen-glucose deprivation in the cortex of GFAP/EGFP or GFAP/EGFP/α-Syn^−/−^ mice. (**A**) Superimposed confocal images of an EGFP-labeled astrocyte in the cortex of a GFAP/EGFP/α-Syn^−/−^ mouse recorded in aCSF (control), during a 20-minute exposure to oxygen-glucose deprivation (OGD) and during a 40-minute washout. The astrocyte volume was quantified after every 5 minutes during OGD and every 20 minutes during washout. (**B left**) Time-dependent changes in the total astrocyte volume in the cortex of GFAP/EGFP (red) and GFAP/EGFP/α-Syn^−/−^ mice (green) during 20-minute OGD and during a 40-minute washout. (**B right**) The changes of astrocyte volume during washout were evaluated in each individual cell and were expressed as an average percent of cell volume increase/decrease related to the maximal volume after 20 minutes of OGD, which was set as 0%. Asterisks indicate very significant (**, p<0.01) differences between GFAP/EGFP and GFAP/EGFP/α-Syn^−/−^ mice.

The participation of the perivascular AQP4 pool in generating edema following ischemia was described *in vivo* in α-Syn^−/−^ mice after middle cerebral artery occlusion [39], [40]. Here, we used 3D-confocal morphometry to clarify how the absence of specifically localized AQP4 may affect astrocyte volume changes evoked by OGD *in situ*. The astrocyte volume changes were quantified during a 20-minute exposure to OGD and following a 40-minute washout. The cell volume was determined every 5 minutes during OGD and every 10 minutes during washout (Fig. 7A). We found that α-syntrophin deletion significantly slows down the OGD-induced astrocytic swelling. While in GFAP/EGFP mice, the astrocytes swelled by 9.0% within the first 5 minutes of OGD, in GFAP/EGFP/α-Syn^−/−^ mice the astrocyte volume remained practically unchanged within first 5 minutes and swelling of 9.0% occurred after 10 minutes (Fig. 7B left). Moreover, after 20 minutes of OGD, GFAP/EGFP astrocytes swelled to 142.4±1.7% (n = 36) of their original volume, while the swelling of GFAP/EGFP/α-Syn^−/−^ astrocytes reached only 131.9±2.1% (n = 33). During washout astrocytes from both GFAP/EGFP and GFAP/EGFP/α-Syn^−/−^ mice continued to swell, and the time course of astrocytic volume changes was comparable (Fig. 7B right).

Taken together, α-syntrophin deletion significantly reduces astrocytic swelling only under severe pathological conditions, such as hypoosmotic stress (205 mOsm/kg), OGD or high extracellular K^+^ (50 mM), presumably when the astrocyte volume rises above ∼120% of the original astrocyte volume. Our data also indicate that α-syntrophin deletion alters the ability of astrocytes to regulate their volume during the washout period following mild stimuli, such as hypoosmotic stress (250 mOsm/kg), 10 mM extracellular K^+^ or hyperosmotic stress.

### Astrocytic soma versus processes

**Figure 8:**
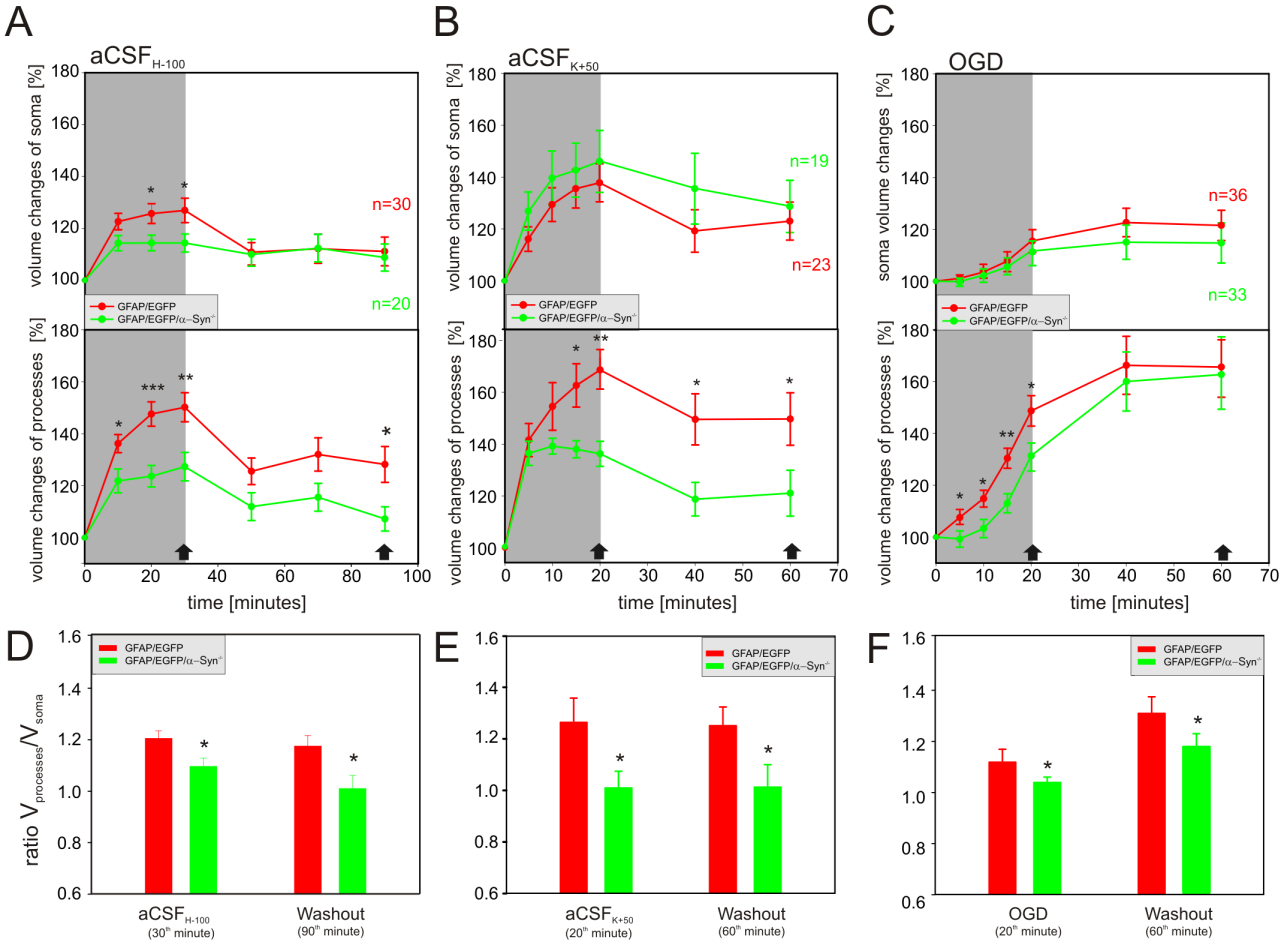
Volume changes in the astrocytic soma and processes during hypotonic stress, increased extracellular K^+^ concentration and OGD. (**A–C**) Time-dependent changes in the volume of the astrocytic soma (**top**) and processes (**bottom**) in GFAP/EGFP (red) and GFAP/EGFP/α-Syn^−/−^ mice (green) during a 30-minute application of aCSF_H-100_ (**A**), a 20-minute application of aCSF_K+50_ (**B**) or 20-minute OGD (**C**), followed by a 60- or 40-minute washout. (**D–F**) The contribution of the astrocytic soma and processes to the total astrocyte volume changes was expressed as a ratio of the volume changes of both compartments (V_processes_/V_soma_) after 30 minutes of hypotonic stress and a subsequent 60-minute washout (**D**), after a 20-minute exposure to aCSF_K+50_ and a subsequent 40-minute washout (**E**), and after 20 minutes of OGD and a subsequent 40-minute washout (**F**). Note that in GFAP/EGFP mice the swelling of the astrocytic processes prevails (V_processes_/V_soma_ = ∼1.2), while in GFAP/EGFP/α-Syn^−/−^ the ratio declines towards 1, indicating that the astrocytic processes swell less and the contribution of the cell soma to total astrocyte volume increases. The time-points at which the ratio was calculated are indicated by arrows in A–C. Asterisks indicate significant (*, p<0.05), very significant (**, p<0.01) and extremely significant (***, p<0.001) differences between GFAP/EGFP and GFAP/EGFP/α-Syn^−/−^ mice.

Considering the deficiency of AQP4s on astrocytic endfeet, we hypothesized that the knockout of α-syntrophin may preferentially result in the reduced swelling of astrocytic processes. Therefore, we quantified volume changes in the astrocytic soma as well as in the processes induced by hypoosmotic stress (205 mOsm/kg), OGD and 50 mM K^+^ in the cortex of GFAP/EGFP mice and compared them to those observed in astrocytes from GFAP/EGFP/α-Syn^−/−^ animals (Fig. 8). The application of aCSF_H-100_ revealed that the swelling of the astrocytic soma and the astrocytic processes was smaller in GFAP/EGFP/α-Syn^−/−^ when compared to GFAP/EGFP mice (Fig. 8A); while during 50 mM K^+^ application and OGD (Fig. 8B, C) the swelling of the astrocytic soma in both groups was comparable, and marked differences were detected only in the astrocytic processes. In GFAP/EGFP/α-Syn^−/−^ mice the swelling of the astrocytic processes was significantly smaller in 50 mM K^+^, and delayed and smaller during OGD than that observed in GFAP/EGFP mice.

In order to estimate the contribution of the cell soma and processes to total astrocyte swelling, we evaluated the changes in astrocytic soma volume (V_soma_) and process volume (V_processes_) as described previously [23] and expressed the volume changes as a ratio (V_processes_/V_soma_) during hypotonic stress, OGD or increased extracellular K^+^ and after washout. This analysis clearly showed that in GFAP/EGFP mice the swelling of the astrocytic processes prevails (V_processes_/V_soma_ = ∼1.2), while in GFAP/EGFP/α-Syn^−/−^ this ratio declines towards a value of 1, indicating that the astrocytic processes swell less and that the contribution of the cell soma to the total astrocyte volume increases (Fig. 8D–F).

### Gene expression profile of astrocytes lacking α-syntrophin

**Figure 9:**
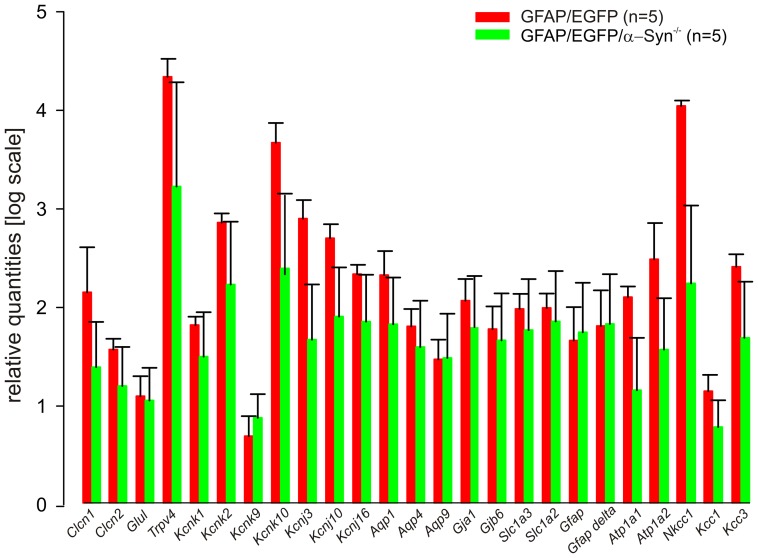
Gene expression profile of cortical astrocytes from GFAP/EGFP and GFAP/EGFP/α-Syn^−/−^ mice. Bar graph indicating the mRNA expression levels for all analyzed genes in 198,400 astrocytes isolated from 5 GFAP/EGFP (red) and 206,640 astrocytes from 5 GFAP/EGFP/α-Syn^−/−^ mice (green); the mRNA levels are presented as the mean ± SEM. *Clcn1* and *Clcn2* code the voltage-gated chloride channels ClC1 and ClC2; *Glul* encodes glutamine synthetase; *Trpv4* encodes the transient receptor potential vanilloid 4 channel TRPV4; *Kcnk1*, *Kcnk2*, *Kcnk9* and *Kcnk10* code the two pore domain K^+^ channels TWIK-1, TREK-1, TASK-3 and TREK2; *Kcnj3*, *Kcnj10* and *Kcnj16* code the inwardly rectifying K^+^ channels Kir3.1, Kir4.1 and Kir5.1; *Aqp1*, *Aqp4* and *Aqp9* code the water channels AQP1, AQP4 and AQP9; *Gja1* and *Gjb6* code connexin43 and connexin30; *Slc1a3* and *Slc1a2* code the glutamate transporters GLAST and GLT-1, *Gfap* and *Gfap-delta* code glial fibrillary acidic protein and its δ splice variant. *Atp1a1* and *Atp1a2* code Na^+^/K^+^-ATPases alpha 1 and 2, *Nkcc1*, *Kcc1* and *Kcc3* code Na-dependent or independent K^+^/Cl^−^ co-transporters.

Besides AQP4 mislocation, α-syntrophin knockout also results in the redistribution of inwardly rectifying K^+^ channels, namely Kir4.1 [8], [9]. Therefore, the question arose whether the lack of α-syntrophin may also lead to changes in the expression of other membrane proteins participating in K^+^ uptake and cell volume regulation. To disclose possible differences between astrocytes from GFAP/EGFP mice and those lacking α-syntrophin, we collected 198,400 EGFP^+^ cells from cortices of GFAP/EGFP mice (n = 5) and 206,640 EGFP^+^ cells from cortices of GFAP/EGFP/α-Syn^−/−^ mice (n = 5) and performed RT-qPCR in both bulk samples. From among all of the 25 analyzed genes (for the list of genes/proteins, see Table 1), in GFAP/EGFP/α-Syn^−/−^ mice slight decreases were observed in the expression of *Kcnk10* (two-pore domain channel TREK-2; p = 0.081), *Nkcc1* (Na^+^/K^+^/Cl^−^ co-transporter; p = 0.053) and *Kcnj3* (inwardly rectifying K^+^ channel Kir3.1; p = 0.071, Fig. 9); however, these changes were not significant. Thus we can conclude that α-syntrophin deletion does not affect the expression of several membrane proteins that participate in astrocyte volume regulation.

## Discussion

In the present study we quantified cell volume changes in astrocytes lacking α-syntrophin thanks to the successful generation of GFAP/EGFP/α-Syn^−/−^ mice, which enabled us to visualize astrocytes based on EGFP expression. Here, we demonstrated that α-syntrophin knockout results in smaller astrocytic swelling when induced by severe hypoosmotic stress, OGD or high extracellular K^+^, and it also affects astrocyte volume changes during the washout period following mild hypo- and hyperosmotic stress, and 10 mMK^+^.

The cortex of the newly generated GFAP/EGFP/α-Syn^−/−^ mice is also characterized by the lack of AQP4 staining in the vicinity of blood vessels as previously described in α-Syn^−/−^ mice [1], [10] and moreover, in response to various stimuli the changes in diffusion parameters *in situ* as well as *in vivo* are similar when compared to those observed in α-Syn^−/−^ mice [12]. The RTI method using TMA^+^ as an extracellular marker was employed in this study to detect possible differences between the newly generated double transgenic strain and the original α-Syn^−/−^ and α-Syn^+/+^ mice with respect to changes in the ECS diffusion parameters in response to pathological stimuli. Our results, such as a higher basal ECS volume both *in situ* and *in vivo*, smaller cell swelling during exposure to more severe stimuli as well as a faster/higher [K^+^]_o_ increase in GFAP/EGFP/α-Syn^−/−^ mice after cardiac arrest, indicate that the tissue and diffusion properties of GFAP/EGFP and GFAP/EGFP/α-Syn^−/−^ mice are indistinguishable from those observed in α-Syn^+/+^ and α-Syn^−/−^ mice, respectively [12]. Therefore, we assume that GFAP/EGFP/α-Syn^−/−^ mice display similar characteristic as α-Syn^+/+^ and α-Syn^−/−^ animals, and that the results obtained by morphometric analysis of astrocytic volume changes are valid for both groups. However, we should always keep in mind that such changes in astrocytic volume and the ECS volume fraction, which reciprocally reflects the volume changes in all cellular elements of the tissue, are not always in direct relationship.

In contrast to the smaller changes in the absolute and relative values of the ECS volume fraction α during terminal ischemia/anoxia *in vivo*, indicating diminished cell swelling in GFAP/EGFP/α-Syn^−/−^ mice, there is a significant increase in tortuosity in these animals when compared to GFAP/EGFP mice. This unexpected finding is most likely associated with the faster and significantly larger increase of [K^+^]_o_ in GFAP/EGFP/α-Syn^−/−^ mice (71.2±1.9 mM, n = 5) in comparison with GFAP/EGFP mice (57.2±2.4 mM, n = 5), probably attributable to insufficient K^+^ buffering. Analyses of the volume changes of the astrocytic soma and processes showed [15] that K^+^-evoked cell swelling affects mostly the astrocytic processes (80%) and that these morphological changes not only affect the final volume of the extracellular space, but also create additional diffusion barriers, thus markedly increasing tortuosity.

It is not that surprising that the application of mild stimuli, such as aCSF_H-50_, aCSF_K+10_ or aCSF_hyper+sucrose_, did not reveal any significant differences in astrocyte swelling or shrinkage between GFAP/EGFP and GFAP/EGFP/α-Syn^−/−^ mice, as these stimuli result in physiological swelling/shrinkage, which may occur as a result of intense neuronal activity. It has been shown that a single action potential can raise local [K^+^]_o_ by as much as 1 mM [41], and repetitive stimulation of a neuronal fiber pathway has been reported to increase [K^+^]_o_ to 12 mM [42], [43]. Comparable astrocyte swelling in both types of transgenic mice also accords well with similar changes in the ECS volume fraction in response to mild hypotonic stress or 10 mM K^+^ (Fig. 2). These findings are also in line with our previous quantification of GFAP immunoreactivity, which revealed no differences in astrocyte morphology in response to mild hypotonic stress or exposure to 10 mM K^+^ between GFAP/EGFP and GFAP/EGFP/α-Syn^−/−^ mice; however, using GFAP staining for morphological evaluation of astrocytes gives only a very rough estimation of astrocytic volume changes. Similarly, ECS volume fraction measurements in Syn^+/+^ and Syn^−/−^ mice showed no differences in the ECS diffusion parameters in response to the above-mentioned mild stimuli [12]. These findings suggest that the contribution of AQP4 channels to water influx under such conditions may be minor or that the influx of water via co-transporters may compensate for the lack of AQP4 in α-syntrophin knockout mice. Since it has been demonstrated that water permeability is not zero in astrocytes from Aqp4-deficient mice [5] and that membrane proteins, such as glutamate transporters or other co-transporters are able to transport water with their substrates [44], it is possible that ion channels/transporters participating in astrocyte swelling/volume regulation during mild stimuli actively transport water. The finding that astrocyte swelling induced by mild stimuli (Fig. 4C, 6C) never exceeded 120% of the original astrocyte volume (100%) reflects physiological astrocyte volume changes as well, because similar astrocyte volume changes (of ∼20%) were observed by Florence and co-authors [45] in response to a physiological increase in extracellular K^+^ concentration of 3 mM.

Of note, the ECS volume fraction in cortical slices from both transgenic mouse strains almost completely returned to basal values during washout following mild hypotonic stress (Fig. 2A), and an even larger ECS volume fraction was detected in the cortex of GFAP/EGFP mice, while astrocytic volume did not return to its original value (100%) in either type of animal. Our findings suggest that changes in ECS volume fraction do not always correlate with astrocyte volume changes and other cellular or even molecular elements contributing to the observed ECS volume recovery should be considered.

In contrast to the physiological swelling of astrocytes, the extent of their swelling induced by severe hypoosmotic stress, the elevation of extracellular K^+^ to 50 mM or OGD revealed significant differences between GFAP/EGFP and GFAP/EGFP/α-Syn^−/−^ mice. Severe hypotonic stress, 50 mM K^+^ and OGD all resulted in smaller astrocytic swelling in the cortex of GFAP/EGFP/α-Syn^−/−^ mice when compared to GFAP/EGFP mice, which also correlates well with the changes in ECS volume fraction observed in GFAP/EGFP/α-Syn^−/−^ mice. It was demonstrated previously that severe hypoosmotic stress results in K^+^ accumulation (∼45 mM K^+^) in the vicinity of astrocytic membrane [33], and thus the smaller swelling of astrocytes of GFAP/EGFP/α-Syn^−/−^ mice might be attributable to the lack of AQP4 on the astrocytic endfeet and the delayed K^+^ clearance described previously [10]. Since AQP4 channels also provide water efflux from astrocytes, the smaller volume changes of astrocytes in the cortex of GFAP/EGFP/α-Syn^−/−^ mice following hypoosmotic stress are probably also caused by AQP4 mislocation.

Despite the fact that astrocyte shrinkage in aCSF_hyper+sucrose_ was similar in both transgenic mouse strains, the volume of astrocytes from GFAP/EGFP mice during washout overshot the original volume, reaching a value of 110%. Since this increase in cell volume, which was shown to be related to the prolonged effect of RVI, was not observed in astrocytes from GFAP/EGFP/α-Syn^−/−^ mice, it probably reflects, besides a smaller influx of water into the cells, alterations in regulatory volume mechanisms. Since it has been shown that AQP4 channels are highly permeable in the presence of high osmotic gradients, the AQP4 mislocation due to the lack of α-syntrophin, might also influence volume changes in aCSF following hyperosmotic stress. Surprisingly, instead of astrocyte shrinkage, hyperosmotic solution containing mannitol (aCSF_hyper+mannitol_) evoked astrocyte swelling, which was smaller in astrocytes from GFAP/EGFP/α-Syn^−/−^ mice compared to EGFP/GFAP mice. Similarly to our data, McManus and co-authors [46] showed that unlike hypertonic saline, hypertonic mannitol exposure produces rebound cell swelling in astroglia. They suggested that the cellular penetration of mannitol appears to account for such a phenomenon.

When compared to GFAP/EGFP mice, the exposure of astrocytes to 50 mM K^+^ revealed smaller, but not delayed swelling of astrocytes in GFAP/EGFP/α-Syn^−/−^ mice. One would expect that delayed astrocyte swelling, presumably due to delayed K^+^ clearance, will occur under these conditions; however, it is possible that the major role in K^+^ uptake is played by Na^+^-dependent K^+^/Cl^−^ co-transporters rather than by Kir4.1 channels [15]. Extracellular K^+^ measurements during the induction of terminal ischemia/anoxia revealed a faster and steeper rise of extracellular K^+^ in GFAP/EGFP/α-Syn^−/−^ mice, which might suggest altered K^+^ clearance, presumably due to changes in the ability of astrocytes to take up K^+^. Employing OGD *in situ*, we showed that astrocyte swelling in GFAP/EGFP/α-Syn^−/−^ mice is delayed, which might also mirror delayed K^+^ clearance. Similar findings of delayed K^+^ clearance in α-syntrophin knockouts were reported following high-frequency synaptic activation [10] or following the activation of neuro-cortical pathways *in vivo* or cortical spreading depression in mice lacking AQP4 [47]. In agreement with Neely and co-authors [8], who showed no changes in Kir4.1 concentration in the astrocytic endfeet of α-syntrophin knockout mice using immunogold analyses, our RT-qPCR analyses of a large number of cortical astrocytes revealed no changes in the expression of genes associated with K^+^/glutamate uptake or cell volume regulation. mRNA levels of AQP4 were not significantly changed, which is in line with the already described AQP4 redistribution from astrocytic endfeet [8]. Surprisingly, we observed no differences in astrocyte volume changes during the washout period following severe hypoosmotic stress, 50 mM K^+^ or OGD. It is highly possible that mild and severe insults activate distinct ion channels/transporters and that therefore, the mechanisms participating in cell volume regulation might differ as well.

**Figure 10:**
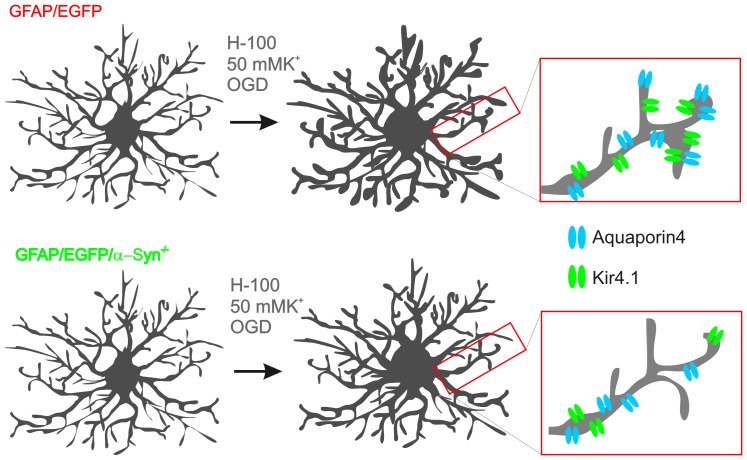
The contribution of processes and cell soma to total astrocyte swelling is altered in astrocytes lacking α-syntrophin. A scheme depicting differences in swelling of astrocytic processes and cell soma in response to severe hypoosmotic stress, 50 mM K^+^ and OGD (oxygen-glucose deprivation), highlighting smaller swelling of astrocytic processes in GFAP/EGFP/α-Syn^−/−^ mice compared to those in GFAP/EGFP mice due to altered distribution of aquaporin4 and Kir4.1 channels.

Our previous study [19] showed that astrocytic processes carry the predominant part of astrocytic swelling, which is in line with our present findings obtained in GFAP/EGFP mice. In contrast, we determined in this work that the average volume changes of the astrocytic soma are very similar to the volume changes of the astrocytic processes in the cortex of GFAP/EGFP/α-Syn^−/−^ mice during exposure to aCSF_H-100_ or aCSF_K+50_, which might point towards AQP4 shift from the astrocytic processes as shown previously by electron microscopy or immunohistochemistry [1], [7], [8], and it might also indicate the translocation of AQP4 towards the cell soma (Figure 10).

Recently, Potokar and co-authors [48] reported that hypoosmotic conditions trigger a transient increase in AQP4 plasma membrane localization, changes of which were related to alterations in AQP4e vesicle trafficking. It is also possible that this increase in plasma membrane AQP4 induced by hypoosmotic stress is attenuated by the lack of α-syntrophin. In agreement with previous findings [8], we did not observe any differences in *Aqp4* expression; however, it is not clear whether AQP4 on the astrocytic membrane is only mislocated, because based on 3D-confocal morphometry (Fig. 8A), we found that under hypoosmotic stress both the astrocytic soma as well as the astrocyte processes in GFAP/EGFP/α-Syn^−/−^ mice swell less than those in GFAP/EGFP mice. A simple relocation of AQP4 from the astrocytic endfeet towards the cell soma would cause comparable or even larger volume changes in the astrocytic soma and thus we hypothesize that AQP4 internalization might occur together with AQP4 mislocation. Moreover, in mice lacking α-syntrophin AQP4 might be differently regulated. However, a comparison of the gene expression profiles of astrocytes from GFAP/EGFP and GFAP/EGFP/α-Syn^−/−^ mice did not reveal any significant alterations in the gene expression profile of astrocytes lacking α-syntrophin.

In our recent papers [15], [19] we have demonstrated the existence of two astrocytic subpopulations in the cortex of GFAP/EGFP mice with distinct abilities to regulate their volume during hypoosmotic stress and in response to OGD. The presence of these subpopulations was also confirmed in the cortex of GFAP/EGFP/α-Syn^−/−^ mice; however, we were unable to detect any significant differences between GFAP/EGFP and GFAP/EGFP/α-Syn^−/−^ mice with respect to the incidence of these subpopulations. Thus, the average astrocytic responses comprise astrocytes of both subpopulations.

The aim of this work was to elucidate the role of α-syntrophin in brain volume regulation under various pathological conditions, which until now has only been studied on the tissue level. Besides the fact that the deletion of α-syntrophin decreases astrocytic swelling under severe pathological conditions, such as hypoosmotic stress, high [K^+^]_o_, or OGD, it also reveals altered regulatory volume mechanisms following hypoosmotic or hyperosmotic stress, or exposure to 10 mM K^+^. Since the influx of water together with ion movement across the cell membrane plays a crucial role in edema formation during ischemic or traumatic brain/spinal cord injuries, further studies focusing on the microdomains in the astrocytic membranes, which may comprise AQP4, K^+^ channels/cotransporters and TRPV4, are essential for understanding the impact of AQP4 mislocation on K^+^ uptake and astrocyte volume regulation.

## Supporting Information

Figure S1 **Fluorescence rundown during repetitive scanning of EGFP-positive cells and fluorescence rundown correction.** (**A**) An example of superimposed images of seven consecutive scanning of a fluorescently labeled astrocyte in the cortex of GFAP/EGFP mice. (**B**) Bar graph indicating the decrease in cell volume during repetitive scanning (black bar) due to photobleaching in astrocyte shown in (A). Gray bar indicates the loss of cell volume due to photobleaching during seven repetitive scanning and the value used for photobleaching correction at. Dotted line highlights the value of an astrocytic volume obtained during first scanning. (**C**) Bar graph indicating astrocyte volume changes during application of hypoosmotic solution without (left) and with correction for photobleaching (right). Note that a decrease in astrocyte volume as a result of repetitive scanning is linear and therefore, the values for correction were estimated from first three repetitive scanning (highlighted in green), prior to application of pathological stimulus. Dotted line indicates the theoretical decrease in astrocyte volume during seven repetitive scanning.
(TIF)

Figure S2 **AQP4 immunoreactivity.** (**A**) Detailed images of AQP4 staining in the cortex of GFAP/EGFP (top) and GFAP/EGFP/α-Syn^−/−^ mice. Note weak AQP4 staining in EGFP-positive astrocyte lacking alpha-syntrophin. (**B**) AQP4 immunostaning in cortical slices isolated from the brain of GFAP/EGFP following slice preparation and also following 90 min incubation in aCSF. Note that there are no changes in AQP4 pattern in the cortex of GFAP/EGFP mice following slice preparation or 90 min aCSF incubation.
(TIF)

Table S1 **Effect of hypotonic stress and elevated K^+^ on the ECS diffusion parameters in GFAP/EGFP and GFAP/EGFP/α-Syn^−/−^ mice** ***in situ*** **.** The values are presented as mean ± S.E.M. Asterisks (***- p<0.001) indicate significant differences between the values in GFAP/EGFP and GFAP/EGFP/α-Syn^−/−^ animals; crosshatches (#- p<0.05; ##- p<0.01;###- p<0.001) indicate significant differences between control values and those obtained under experimental conditions in the same group of animals. The control values of the ECS diffusion parameters from each individual experiment were calculated as the average values extracted from three diffusion curves before application. The mean control value presented in the table is the average of the control values from all *in vitro* experiments. The mean values of the maximum change during application correspond to the 30^th^ minute of application; the values for washout correspond to the data point at the 90^th^ min. Abbreviations: extracellular space volume fraction (α), tortuosity (λ), non-specific uptake (*k′*), number of animals (N), number of slices (n).
(DOCX)

Table S2 **Effect of terminal ischemia/anoxia on the ECS diffusion parameters and extracellular K^+^ concentration in GFAP/EGFP and GFAP/EGFP/α-Syn^−/−^ mice** ***in vivo*** **.** The values are presented as mean ± S.E.M. Asterisks (*- p<0.05; ***- p<0.001) indicate significant differences between the values in GFAP/EGFP and GFAP/EGFP/α-Syn^−/−^ animals; crosshatches (###- p<0.001) indicate significant differences between control values and those obtained under experimental conditions in the same group of animals.
(DOCX)
