# Correction: Altered Astrocytic Swelling in the Cortex of α-Syntrophin-Negative GFAP/EGFP Mice

**DOI:** 10.1371/journal.pone.0119744

**Published:** 2015-03-16

**Authors:** The PLOS ONE Staff

In the Funding section, the following grant number from the Grant Agency of the Czech Republic is missing: P303-11-2378. The correct, complete funding information is as follows: This study was supported by the grants GA CR 13-02154S, P303-11-2378 and P304/12/G069 from the Grant Agency of the Czech Republic, by the project BIOCEV CZ.1.05/1.1.00/02.0109 from the European Regional Development Fund and by the Operational Programme Education for Competitiveness CZ.1.07/2.3.00/30.0045 from the European Social Fund and the state budget of the Czech Republic. The authors state that the funders had no role in study design, data collection and analysis, decision to publish, or preparation of the manuscript.
