# Supplementary figures and images for: Altered Astrocytic Swelling in the Cortex of α-Syntrophin-Negative GFAP/EGFP Mice
Source: PLoS One. 2014 Nov 26;9(11):e113444. doi: 10.1371/journal.pone.0113444 (PMC4245134; doi:10.1371/journal.pone.0113444)

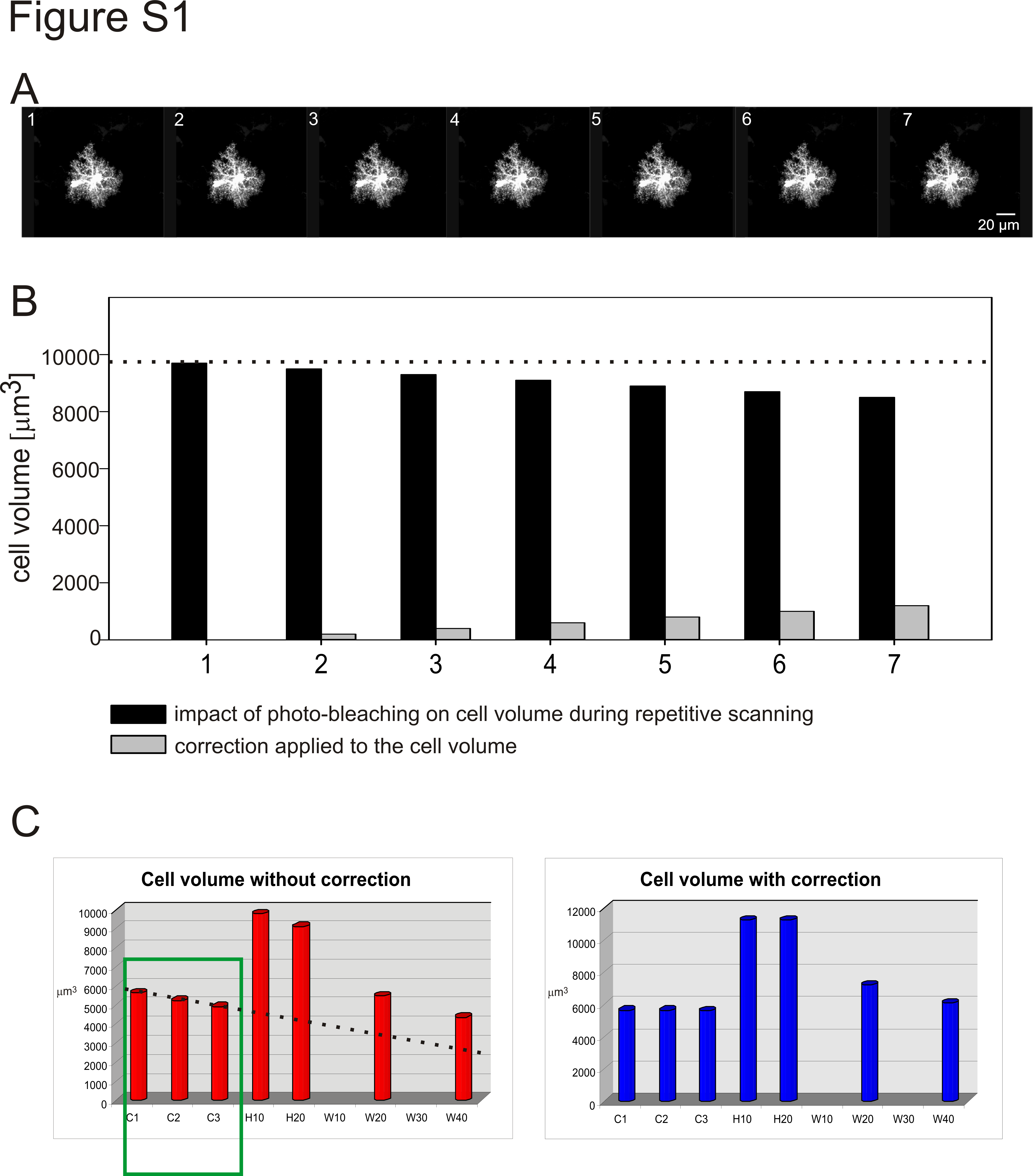

Supplement: Figure S1 — Fluorescence rundown during repetitive scanning of EGFP-positive cells and fluorescence rundown correction. (A) An example of superimposed images of seven consecutive scanning of a fluorescently labeled astrocyte in the cortex of GFAP/EGFP mice. (B) Bar graph indicating the decrease in cell volume during repetitive scanning (black bar) due to photobleaching in astrocyte shown in (A). Gray bar indicates the loss of cell volume due to photobleaching during seven repetitive scanning and the value used for photobleaching correction at. Dotted line highlights the value of an astrocytic volume obtained during first scanning. (C) Bar graph indicating astrocyte volume changes during application of hypoosmotic solution without (left) and with correction for photobleaching (right). Note that a decrease in astrocyte volume as a result of repetitive scanning is linear and therefore, the values for correction were estimated from first three repetitive scanning (highlighted in green), prior to application of pathological stimulus. Dotted line indicates the theoretical decrease in astrocyte volume during seven repetitive scanning. (TIF) [file pone.0113444.s001.tif]

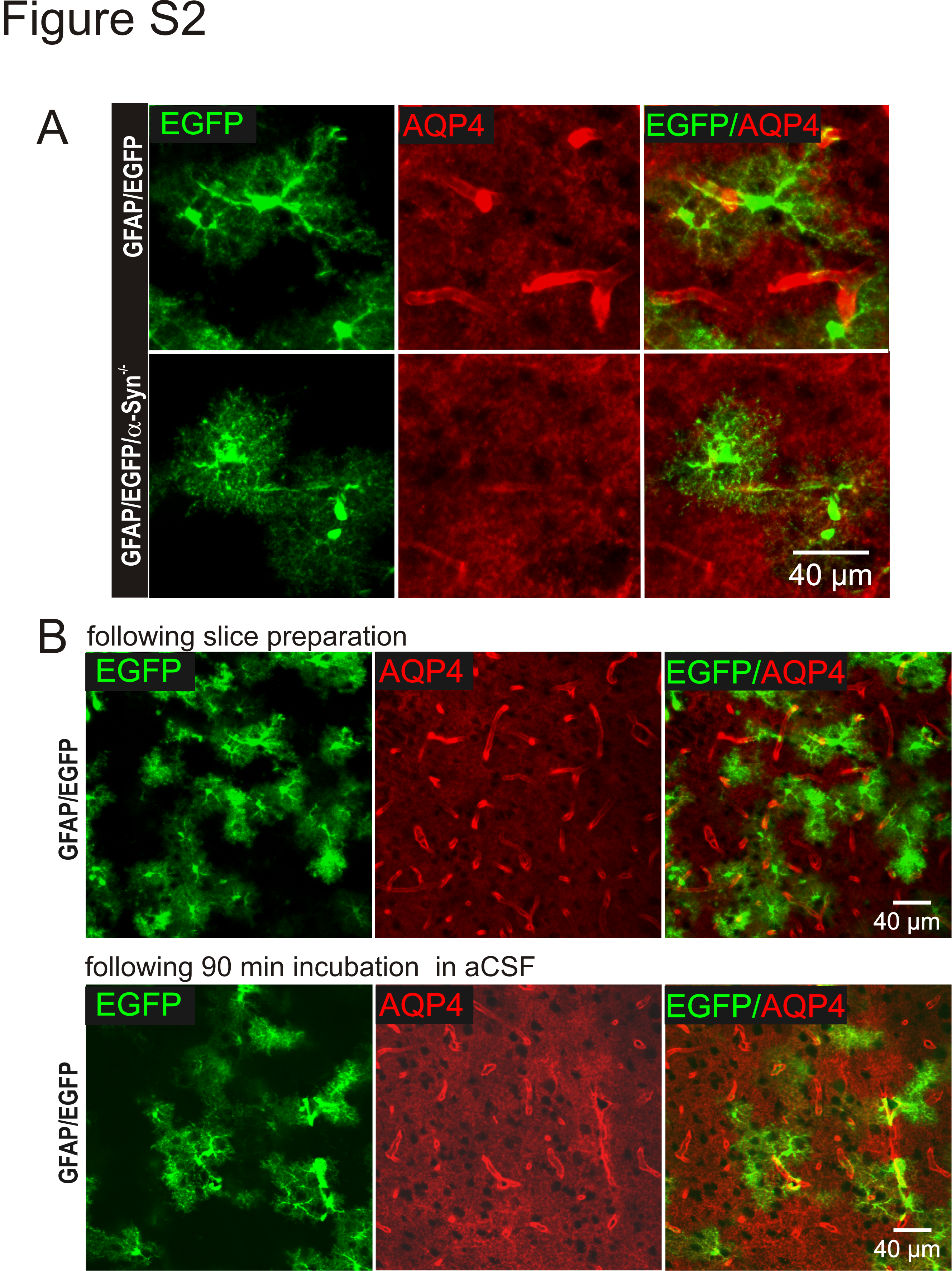

Supplement: Figure S2 — AQP4 immunoreactivity. (A) Detailed images of AQP4 staining in the cortex of GFAP/EGFP (top) and GFAP/EGFP/α-Syn−/− mice. Note weak AQP4 staining in EGFP-positive astrocyte lacking alpha-syntrophin. (B) AQP4 immunostaning in cortical slices isolated from the brain of GFAP/EGFP following slice preparation and also following 90 min incubation in aCSF. Note that there are no changes in AQP4 pattern in the cortex of GFAP/EGFP mice following slice preparation or 90 min aCSF incubation. (TIF) [file pone.0113444.s002.tif]
