# Supplementary material for: Altered Astrocytic Swelling in the Cortex of α-Syntrophin-Negative GFAP/EGFP Mice
Source: PLoS One. 2014 Nov 26;9(11):e113444. doi: 10.1371/journal.pone.0113444 (PMC4245134; doi:10.1371/journal.pone.0113444)
Supplement: Table S1 — Effect of hypotonic stress and elevated K+ on the ECS diffusion parameters in GFAP/EGFP and GFAP/EGFP/α-Syn−/− mice in situ . The values are presented as mean ± S.E.M. Asterisks (***- p<0.001) indicate significant differences between the values in GFAP/EGFP and GFAP/EGFP/α-Syn−/− animals; crosshatches (#- p<0.05; ##- p<0.01;###- p<0.001) indicate significant differences between control values and those obtained under experimental conditions in the same group of animals. The control values of the ECS diffusion parameters from each individual experiment were calculated as the average values extracted from three diffusion curves before application. The mean control value presented in the table is the average of the control values from all in vitro experiments. The mean values of the maximum change during application correspond to the 30th minute of application; the values for washout correspond to the data point at the 90th min. Abbreviations: extracellular space volume fraction (α), tortuosity (λ), non-specific uptake (k′), number of animals (N), number of slices (n). (DOCX) [file pone.0113444.s003.docx]

**Table S1**

| **GFAP/EGFP** | **α** | **λ** | ***k´* (10^-3^s^-1^)** | **n/N** |
| --- | --- | --- | --- | --- |
| control values | 0.189 ± 0.002 | 1.514 ± 0.011 | 11.115 ± 1.004 | 26/16 |
| aCSF_H-50_ | 0.149 ± 0.007^###^ | 1.553 ± 0.031 | 10.465 ± 1.756 | 6/4 |
| washout in aCSFt | 0.215 ± 0.011^#^ | 1.558 ± 0.023 | 9.318 ± 1.186 | 6/4 |
| aCSF_H-100_ | 0.089 ± 0.012^###^ | 1.559 ± 0.068 | 13.955 ± 2.581 | 7/4 |
| washout in aCSF | 0.205 ± 0.005 | 1.565 ± 0.072 | 10.449 ± 1.655 | 7/4 |
| aCSF_K+10_ | 0.087 ± 0.018^###^ | 1.582 ± 0.034^#^ | 14.316 ± 2.493 | 7/5 |
| washout in aCSF | 0.218 ± 0.012^##^ | 1.583 ± 0.050 | 10.972 ± 1.649 | 7/5 |
| **GFAP/EGFP/α-Syn ^-/-^** | **α** | **λ** | **k´ (10-3s-1)** | **n/N** |
| control values | 0.217 ± 0.003^***^ | 1.523 ± 0.010 | 9.023 ± 0.731 | 32/22 |
| aCSF_H-50_ | 0.168 ± 0.012^###^ | 1.582 ± 0.033 | 12.242 ± 0.939 | 5/4 |
| washout in aCSFt | 0.211 ± 0.026 | 1.601 ± 0.029^#^ | 10.452 ± 1.025 | 5/4 |
| aCSF_H-100_ | 0.156 ± 0.009^***###^ | 1.557 ± 0.059 | 9.795 ± 1.795 | 7/7 |
| washout in aCSF | 0.274 ± 0.019^***###^ | 1.532 ± 0.043 | 8.191 ± 1.631 | 7/7 |
| aCSF_K+10_ | 0.161 ± 0.012^***###^ | 1.590 ± 0.027 | 10.620 ± 1.766 | 7/6 |
| washout in aCSF | 0.205 ± 0.011 | 1.558 ± 0.023 | 8.943 ± 1.222 | 7/6 |
