# Supplementary material for: Altered Astrocytic Swelling in the Cortex of α-Syntrophin-Negative GFAP/EGFP Mice
Source: PLoS One. 2014 Nov 26;9(11):e113444. doi: 10.1371/journal.pone.0113444 (PMC4245134; doi:10.1371/journal.pone.0113444)
Supplement: Table S2 — Effect of terminal ischemia/anoxia on the ECS diffusion parameters and extracellular K+ concentration in GFAP/EGFP and GFAP/EGFP/α-Syn−/− mice in vivo . The values are presented as mean ± S.E.M. Asterisks (*- p<0.05; ***- p<0.001) indicate significant differences between the values in GFAP/EGFP and GFAP/EGFP/α-Syn−/− animals; crosshatches (###- p<0.001) indicate significant differences between control values and those obtained under experimental conditions in the same group of animals. (DOCX) [file pone.0113444.s004.docx]

**Table S2**

| **GFAP/EGFP** | **control values** | **N** | **terminal ischemia/anoxia** | **N** |
| --- | --- | --- | --- | --- |
| α | 0.193 ± 0.002 | 6 | 0.122 ± 0.010^###^ | 6 |
| λ | 1.600 ± 0.014 | 6 | 1.893 ± 0.026^###^ | 6 |
| *k´*(10^-3^ s^-1^) | 4.309 ± 0.295 | 6 | 6.607 ± 3.018 | 6 |
| [K^+^]_e_ (mM) | 2.847 ± 0.052 | 6 | 57.180 ± 2.426^###^ | 5 |
| **GFAP/EGFP/α-Syn^-/-^** | **control values** | **N** | **terminal ischemia/anoxia** | **N** |
| α | 0.224 ± 0.004^***^ | 6 | 0.164 ± 0.006^*###^ | 6 |
| λ | 1.640 ± 0.012 | 6 | 2.262 ± 0.086^*###^ | 6 |
| *k´*(10^-3^ s^-1^) | 3.522 ± 0.447 | 6 | 5.779 ± 1.570 | 6 |
| [K^+^]_e_ (mM) | 2.877 ± 0.116 | 6 | 71.210 ± 1.879^###^ | 5 |
